# Biallelic *CACNA2D1* loss-of-function variants cause early-onset developmental epileptic encephalopathy

**DOI:** 10.1093/brain/awac081

**Published:** 2022-03-16

**Authors:** Shehrazade Dahimene, Leonie von Elsner, Tess Holling, Lauren S Mattas, Jess Pickard, Davor Lessel, Kjara S Pilch, Ivan Kadurin, Wendy S Pratt, Igor B Zhulin, Hongzheng Dai, Maja Hempel, Maura R Z Ruzhnikov, Kerstin Kutsche, Annette C Dolphin

**Affiliations:** Department of Neuroscience Physiology and Pharmacology, University College London (UCL), London WC1E 6BT, UK; Institute of Human Genetics, University Medical Center Hamburg-Eppendorf, 20246 Hamburg, Germany; Institute of Human Genetics, University Medical Center Hamburg-Eppendorf, 20246 Hamburg, Germany; Neurology and Neurological Sciences, Pediatrics, Division of Medical Genetics, Stanford University and Lucile Packard Children's Hospital, Palo Alto, CA 94304, USA; Department of Neuroscience Physiology and Pharmacology, University College London (UCL), London WC1E 6BT, UK; Institute of Human Genetics, University Medical Center Hamburg-Eppendorf, 20246 Hamburg, Germany; Department of Neuroscience Physiology and Pharmacology, University College London (UCL), London WC1E 6BT, UK; Department of Neuroscience Physiology and Pharmacology, University College London (UCL), London WC1E 6BT, UK; Department of Neuroscience Physiology and Pharmacology, University College London (UCL), London WC1E 6BT, UK; Department of Microbiology and Translational Data Analytics Institute, The Ohio State University, Columbus, OH, 43210, USA; Department of Molecular and Human Genetics, Baylor College of Medicine/NGS-Molecular, Baylor Genetics, Houston, TX, USA; Institute of Human Genetics, University Medical Center Hamburg-Eppendorf, 20246 Hamburg, Germany; Neurology and Neurological Sciences, Pediatrics, Division of Medical Genetics, Stanford University and Lucile Packard Children's Hospital, Palo Alto, CA 94304, USA; Institute of Human Genetics, University Medical Center Hamburg-Eppendorf, 20246 Hamburg, Germany; Department of Neuroscience Physiology and Pharmacology, University College London (UCL), London WC1E 6BT, UK

**Keywords:** epileptic encephalopathy, calcium channel, loss-of-function, biallelic variants, *CACNA2D1*

## Abstract

Voltage-gated calcium (Ca_V_) channels form three subfamilies (Ca_V_1–3). The Ca_V_1 and Ca_V_2 channels are heteromeric, consisting of an α_1_ pore-forming subunit, associated with auxiliary Ca_V_β and α_2_δ subunits. The α_2_δ subunits are encoded in mammals by four genes, *CACNA2D1*–*4*. They play important roles in trafficking and function of the Ca_V_ channel complexes. Here we report biallelic variants in *CACNA2D1*, encoding the α_2_δ-1 protein, in two unrelated individuals showing a developmental and epileptic encephalopathy. Patient 1 has a homozygous frameshift variant c.818_821dup/p.(Ser275Asnfs*13) resulting in nonsense-mediated mRNA decay of the *CACNA2D1* transcripts, and absence of α_2_δ-1 protein detected in patient-derived fibroblasts. Patient 2 is compound heterozygous for an early frameshift variant c.13_23dup/p.(Leu9Alafs*5), highly probably representing a null allele and a missense variant c.626G>A/p.(Gly209Asp). Our functional studies show that this amino-acid change severely impairs the function of α_2_δ-1 as a calcium channel subunit, with strongly reduced trafficking of α_2_δ-1^G209D^ to the cell surface and a complete inability of α_2_δ-1^G209D^ to increase the trafficking and function of Ca_V_2 channels. Thus, biallelic loss-of-function variants in *CACNA2D1* underlie the severe neurodevelopmental disorder in these two patients. Our results demonstrate the critical importance and non-interchangeability of α_2_δ-1 and other α_2_δ proteins for normal human neuronal development.

## Teaser

Dahimene *et al.* report biallelic pathogenic variants in *CACNA2D1*, encoding a calcium channel subunit α2δ-1, in two unrelated individuals with developmental epileptic encephalopathy. One has a homozygous frameshift variant; the other is compound heterozygous for a frameshift and a missense variant rendering α2δ-1 non-functional.

## Introduction

Voltage-gated calcium (Ca_V_) channels are present in all excitable cells including neurons, and open following membrane depolarization, allowing Ca^2+^ entry.^1^ The α1 pore-forming subunits are encoded by a family of 10 mammalian genes, divided into three subfamilies (Ca_V_1–3).^1^ In neurons, Ca_V_2 channels are mainly presynaptic and involved in synaptic transmission.^2^

The Ca_V_1 and Ca_V_2 subfamilies are associated with auxiliary Ca_V_β and α_2_δ subunits.^2^ The α_2_δ proteins are encoded by four mammalian genes, *CACNA2D1–4*,^2^ encoding an α_2_δ pre-protein that is post-translationally cleaved into two polypeptides, α_2_ and δ.^3^ These extracellular glycoproteins remain disulphide-bonded together, linked into the plasma membrane by a glycosylphosphatidyl-inositol anchor.^4^ Human α_2_δ-1 pre-protein (P54289, UniProt) has 1103 amino acids. In conjunction with β, the α_2_δ subunits play important roles in trafficking and function of Ca_V_1 and Ca_V_2 channels.^5^

Evidence that α_2_δ proteins are involved in neurological disease has been reviewed recently.^6^ Initial evidence was for α_2_δ-2, and came from the spontaneous *Cacna2d2* mouse mutant strains including *ducky*,^7^ which demonstrate absence epilepsy and severe cerebellar ataxia when both alleles are mutated. This reflects the strong expression of α_2_δ-2 in particular neurons, specifically cerebellar Purkinje cells.^7^ In humans, biallelic *CACNA2D2* variants cause a phenotypic spectrum ranging from congenital ataxia with cerebellar vermian atrophy on brain imaging^8^ to cerebellar atrophy and developmental and epileptic encephalopathies (DEEs).^9,10^ DEEs are characterized by intractable seizures and developmental impairment or regression.^11^

Here we report two unrelated patients with biallelic *CACNA2D1* variants, one with a homozygous frameshift variant and the other with compound heterozygosity for an early frameshift and a missense variant. Both individuals show a highly consistent phenotype, corresponding to DEE. We demonstrate that the homozygous frameshift variant causes α_2_δ-1 loss in patient fibroblasts, and investigate the effect of the α_2_δ-1 amino-acid change p.(Gly209Asp) on calcium channel function. Our data indicate that biallelic loss-of-function variants in *CACNA2D1* underlie this DEE.

## Materials and methods

### Patients

Informed consent for genetic analyses was obtained for the two patients. Genetic studies were performed clinically or as approved by the Institutional Review Boards of the relevant institutions (Ethics Committee, Hamburg Medical Chamber; PV3802). The patients’ parents provided written informed consent for study participation, clinical data and specimen collection, genetic analysis and publication of relevant findings.

### Exome sequencing, analysis and variant validation

Genomic DNA was extracted from peripheral blood samples using standard procedures. We performed trio exome sequencing with DNA samples of Patient 1 and both healthy parents (Supplementary material). Primer sequences are in Supplementary Table 1. For Patient 2, trio exome sequencing was undertaken with DNA samples of the proband and both healthy parents at Baylor Genetics (Supplementary material).

### RNA isolation and transcript analysis

RNA isolation from fibroblasts, complementary DNA synthesis, polymerase chain reaction (PCR) and Sanger sequencing of amplicons to analyse *CACNA2D1* transcripts were performed as described.^12^ Quantitative PCR to determine the relative mRNA levels of *CACNA2D1* and *CACNA2D3* was performed as described.^12^ Primer sequences are in Supplementary Table 1.

### Bioinformatic analysis of CACNA2D1 homologues

Details of bioinformatic analysis are described in the Supplementary material.

### Cell culture and antibodies

Primary fibroblasts, tsA-201 cells and hippocampal neurons were cultured as described (Supplementary material). The antibodies used are described in Supplementary material.

### Immunoblot analysis of fibroblasts

Whole-cell lysates from patient and control fibroblasts were prepared, and immunoblotting performed as described^12^ (Supplementary material).

### Expression constructs, mutagenesis and cell transfection

Details of expression constructs, transfection markers and procedures are detailed in Supplementary material. The tsA-201 cells were transfected with PolyJet or Fugene6 (as stated) according to manufacturers’ protocols. Hippocampal neurons were transfected with Lipofectamine 2000 (Life Technologies).

### Electrophysiology

Ca_V_2.1 and Ca_V_2.2 currents in transfected tsA-201 cells were investigated by whole-cell patch-clamp recording (Supplementary material).

### Cell surface biotinylation, co-immunoprecipitation and immunocytochemistry

Cell surface biotinylation, co-immunoprecipitation, immunoblotting and immunocytochemistry was performed as described in Kadurin *et al*.^13^ (Supplementary material).

### Data analysis and availability

Data analysis and availability are described in the Supplementary material.

## Results

### Biallelic variants in *CACNA2D1* in two unrelated individuals with DEE

**Figure 1:**
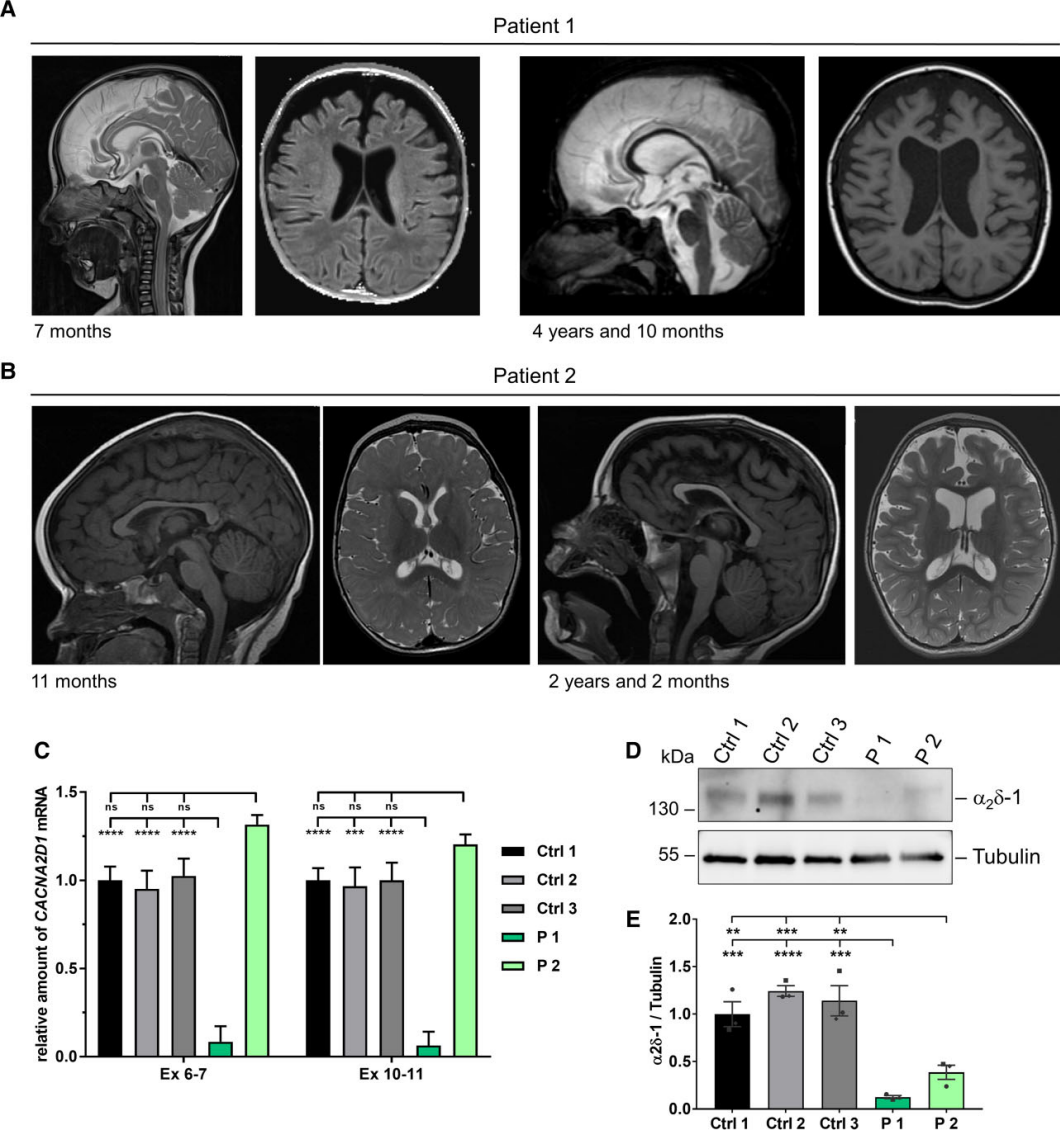
**MRI scans of patients with biallelic variants in *CACNA2D1* and determination of *CACNA2D1* mRNA and α_2_δ-1 protein levels in patient-derived fibroblasts.** (**A**) In Patient 1, T_2_-weighted sagittal MRI shows hypoplastic corpus callosum and T_1_-weighted axial image shows enlarged ventricles and frontotemporal CSF spaces at the age of 7 months. At the age of 4 years and 10 months, progressive frontotemporal and mesial temporal atrophy was noted. (**B**) At the age of 11 months, T_1_-weighted sagittal and T_2_-weighted axial MRI images of Patient 2 show non-specific findings of delayed myelination within the frontal and parietal white matter for age and prominent perivascular spaces. At the age of 26 months, T_1_-weighted sagittal MRI shows interval generalized volume loss with extra-axial spaces and thinning of the corpus callosum. T_2_-weighted axial images show an increase in ventricular size in addition to increased intra-axial spaces. Permission to publish MRI scans was provided for the two patients shown here. (**C**) Relative quantification of *CACNA2D1* transcripts by reverse transcription-qPCR (RT-qPCR) using two *CACNA2D1*-specific primer pairs generating amplicons for exons 6–7 and 10–11. RNA was obtained from fibroblasts of Patients 1 and 2 and three healthy individuals (Controls 1–3). Glyceraldehyde-3-phosphate dehydrogenase (*GAPDH*) mRNA was used as an internal control; and the amount of target mRNA relative to *GAPDH* mRNA is presented. Mean ±  standard error of the mean (SEM) of three independent experiments, each performed in triplicate, is shown. One-way ANOVA with Bonferroni *post hoc* test for multiple comparisons was used for statistical analysis: ns, *P* > 0.05; ***P = 0.0001; ****P < 0.0001. Datasets of independent RT-qPCR experiments with technical triplicates are shown in Supplementary Fig. 2. (**D**) Representative immunoblot of whole-cell lysates obtained from fibroblasts of Patients 1 and 2 and three controls. The amount of α_2_δ-1 was monitored with an anti-α_2_δ-1 antibody. An anti-tubulin antibody was used to demonstrate equal loading. A band corresponding to α_2_δ-1, which shows a low expression in fibroblasts, was observed in all control cells. Uncropped blots are shown in Supplementary Fig. 3. (**E**) Band intensities were quantified using the ChemiDoc imaging system. Mean ± SEM with individual data-points of three independent experiments is shown. One-way ANOVA followed by a Bonferroni *post hoc* test for multiple comparisons was used for statistical analysis: **P < 0.0065; ***P < 0.0005; ****P < 0.0001. Ctrl = control; ex = exon; ns = not significant; P = patient.

Through GeneMatcher,^14^ we identified the two unrelated male individuals, Patients 1 and 2, with a highly consistent phenotype corresponding to DEE. The two affected individuals carried biallelic pathogenic variants in *CACNA2D1* (Tables 1 and 2). Patient 1 developed generalized seizures at age 19 months, while onset of epilepsy was at age 11.5 months in Patient 2. At last examination, Patient 1 was 4 years 11 months and Patient 2 was 4 years old. Both were microcephalic, had severe hypotonia, absent speech, spasticity, choreiform movements, orofacial dyskinesia and cortical visual impairment (Tables 1 and 2, and case reports in the Supplementary material). Brain imaging revealed corpus callosum hypoplasia and progressive volume loss in both (Fig. 1A and B). Patient 1 had no cardiac anomalies, while a tiny patent foramen ovale was found in Patient 2 (Tables 1 and 2).

**Table 1:** Family history, growth parameters and manifestation of first symptoms in patients with biallelic *CACNA2D1* variants

	Patient 1	Patient 2
Ethnicity	Afghan	Caucasian, Native American
Parental consanguinity	First cousins	No
Family history	Epileptic encephalopathy in paternal uncle	No
Sex	Male	Male
*CACNA2D1* variants (NM_000722.3)	c.818_821dup/p.(Ser275Asnfs*13) homozygous	c.13_23dup/p.(Leu9Alafs*5) and c.626G>A/p.(Gly209Asp) compound heterozygous
Pregnancy	Uncomplicated	Uncomplicated
Birth at	37 weeks, uncomplicated	40 weeks
Birth weight, g/*z*-score	2960/−0.5	3345/−0.73
Birth length, cm/*z*-score	50/−0.2	Unknown
OFC at birth, cm/*z*-score	33/−1.0	Unknown
Age at last examination	4 years 11 months	4 years
Weight at last examination, kg/*z*-score	18/−0.4	15.5/−0.52
Length at last examination, cm/*z*-score	110/−0.2	97.8/−0.68
OFC at last examination, cm/*z*-score	48.9/−2.1	47.5/−2.02
**Manifestation**		
First symptoms at age of	3 months	<2 months
First clinical signs	Severe hypotonia with poor head control, no visual attention	Hypotonia with poor head control, decreased visual attention

**Table 2:** Neurological and other findings in patients with biallelic *CACNA2D1* variants

Neurological signs		
Global developmental delay	Profound	Profound
Motor skills	No achievement of motor milestones	Rolls to one side, reaches for toys intermittently
Muscular hypotonia	Severe axial, insufficient head control	Markedly low axial tone, appendicular tone is also low, increased with activation
Spasticity	In all extremities, starting at 2 years	Hands often fisted since birth, spastic catch at elbows and knees more prominent over time, back arching episodes beginning <12 months
Dystonic movements	Choreiform movements of upper extremities, orofacial dyskinesia, onset <2 years	Distal choreiform movements of all extremities, orofacial dyskinesia and generalized dystonic episodes, onset <10 months
Intellectual disability	Profound	Profound
Speech impairment	Profound	Profound
Behaviour	No concerns	No concerns
Cerebral MRI	Hypoplastic corpus callosum, enlarged inner and outer CSF spaces at age 7 months; progressive frontotemporal and mesial temporal atrophy at 4 years 10 months	Generalized volume loss, borderline thinning of the corpus callosum at 11 months; progression of generalized volume loss at 26 months
Hearing	Normal, not tested	Normal
Eyes	Cortical visual impairment, nystagmus	Cortical visual impairment, intermittent disconjugate gaze
**Seizures**		
Age of onset	9 months: absences; 19 months: generalized seizures	11 months: right face/arm twitching, abnormal EEG
Initial seizure type	Absences, generalized	Focal with impaired awareness (hemi-clonic), atypical absence
Current seizure type	Absences	Focal with impaired awareness (hemi-clonic), 3 years 9 months: ESES
Response to treatment	Well (generalized seizures), poor (absences)	Well controlled on Depakene
EEG	Normal at age 7 months	11 months: diffusely slow, no anterior posterior frequency amplitude gradient, slow spike and wave in sleep, multifocal spikes. Focal motor seizure captured, some irregular generalized spikes with possible eye blinking and unresponsive. 3 years 9 months: mild diffuse slowing, occasional multifocal sharps and 2–3 Hz spike and wave during wakefulness, near continuous slow spike and wave during sleep meeting criteria for ESES
**Feeding**		
Feeding difficulties	G tube placement at 4 years	Since birth, s/p G tube placement at 13 months
**Cardiac features**		
Echocardiography	Normal	Limited study with tiny patent foramen ovale, normal function
Electrocardiography	Normal	Normal, including 24 h patch recording
**Other findings**		
Facial dysmorphism	Bitemporal narrowing, large ears, flared medial eyebrows, open mouth with tented upper lip	Microcephalic with posterior plagiocephaly, mild bitemporal narrowing, ears are low set and appear larger relative to head size, mild ptosis bilaterally, mouth held hanging open, high arched palate, teeth are widely spaced and blunted due to frequent bruxism, small hands and feet
Sleep disturbance	Yes	Obstructive sleep apnoea
Insensibility to pain	Yes	Yes

ESES = electrical status epilepticus in sleep; s/p = status post.

Trio exome sequencing in Patient 1 and parents revealed in the proband the homozygous *CACNA2D1* (NM_000722.3) frameshift variant c.818_821dupGAAC/p.(Ser275Asnfs*13). The variant is absent from public databases including gnomAD (v.2.1.1 and 3.1.1). The 4-bp duplication was validated in Patient 1’s DNA and fibroblast-derived complementary DNA. His healthy parents were heterozygous carriers (Tables 1 and 2 and Supplementary Fig. 1). In Patient 2, trio exome sequencing demonstrated compound heterozygosity for the *CACNA2D1* variants c.13_23dupTGCCTGCTGGC [p.(Leu9Alafs*5)] and c.626G>A [p.(Gly209Asp)] (Tables 1 and 2 and Supplementary Fig. 1). His healthy mother was heterozygous for the c.13_23dupTGCCTGCTGGC variant and his healthy father for the c.626G>A variant. The 11-bp duplication is probably a loss-of-function variant; it has a worldwide minor allele frequency of 0.003% (gnomAD v.2.1.1), while the c.626G>A variant is absent in gnomAD (v.2.1.1 and 3.1.1). The missense variant c.626G>A/p.(Gly209Asp) in exon 7 is predicted to be damaging by *in silico* tools detailed in the Supplementary material.

In fibroblasts of Patient 1, *CACNA2D1* mRNA level was reduced to 6–9% compared with control fibroblasts, while it was similar in Patient 2 and control fibroblasts (Fig. 1C and Supplementary Fig. 2). We next determined α_2_δ-1 levels in whole-cell lysates from cultured primary fibroblasts of Patients 1 and 2. Qualitatively, we detected little full-length α_2_δ-1 in Patient 1 fibroblasts, while it was present in Patient 2 and control cells (Fig. 1D and Supplementary Fig. 3). Quantification of α_2_δ-1 indicated 10–12% in Patient 1 and 31–38% in Patient 2 compared to controls (Fig. 1E). Together, these data indicate that Patient 1 carries biallelic *CACNA2D1* loss-of-function alleles and Patient 2 harbours at least one *CACNA2D1* loss-of-function variant.

Next, we investigated mRNA levels of the other *CACNA2D* genes in patient fibroblasts to identify possible compensatory effects. While mRNA levels of *CACNA2D2* and *CACNA2D4* were too low to be quantified, we detected 3- to 7-fold higher *CACNA2D3* mRNA levels in Patient 2 fibroblasts compared to Patient 1 and control cells (Supplementary Fig. 4).

### Glycine 209 is invariant in CACNA2D1

The α_2_δ-1 protein (also denoted as CACNA2D1) contains a von Willebrand factor-A domain and four Ca^2+^ channel and chemotaxis receptor (Cache) domains,^15^ organized into two double-Cache (dCache) domains.^16^ The p.(Gly209Asp) (G209D) amino-acid substitution in the CACNA2D1 gene product of Patient 2 is within the gabapentin and amino-acid binding pocket of its dCache_1 domain.^16^ This Gly residue is important for maintaining a three-strand beta-sheet stability and simultaneously providing a critical turn in the structure. G209 is absolutely invariant in both CACNA2D1 and CACNA2D2 orthologues in all vertebrates and paralogues and predecessors from low invertebrates (Supplementary Fig. 5).

### The p.(Gly209Asp) variant disrupts plasma membrane α_2_δ-1 expression

**Figure 2:**
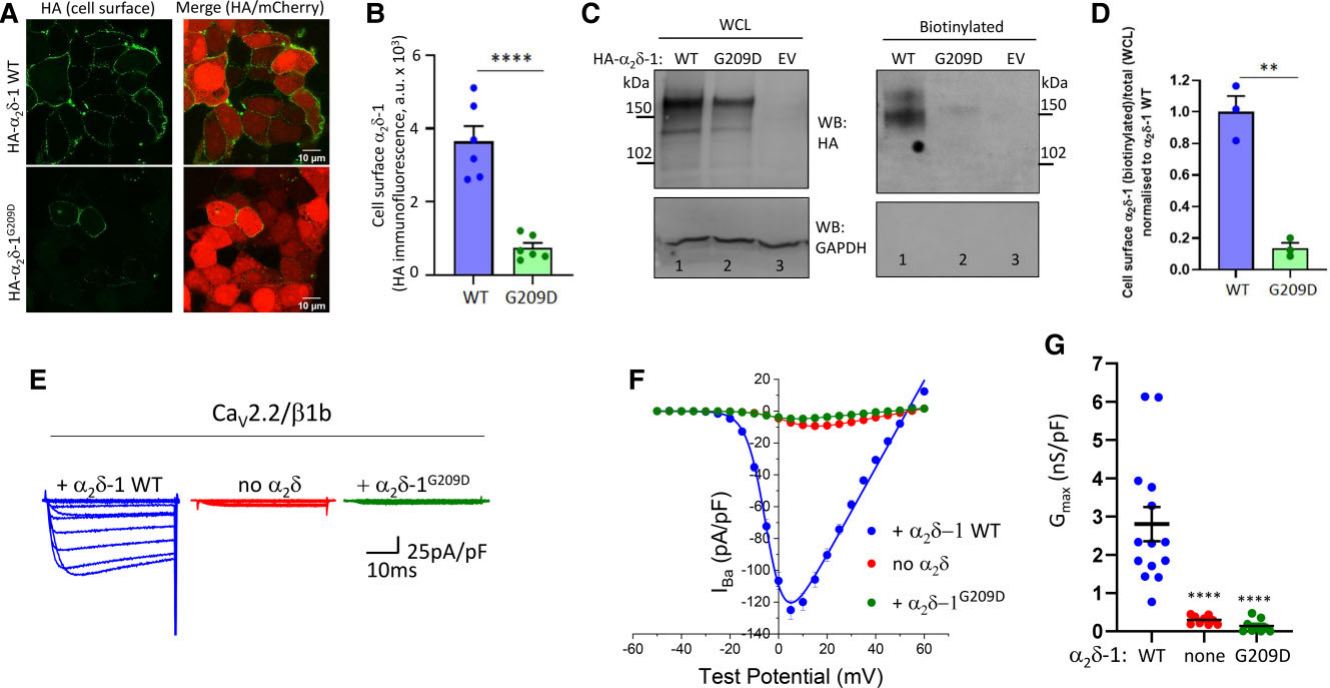
**The p.(Gly209Asp) variant disrupts α_2_δ-1 expression at the cell surface**, **and does not promote Ca_V_2 calcium currents.** (**A**) Representative confocal images of tsA-201 cells transfected with either HA-α_2_δ-1 wild-type (WT) or HA-α_2_δ-1^G209D^ with mCherry. HA staining was performed in non-permeabilized conditions and shown in the *left* panels. The *right* panels represent the merged images with the 10 μm scale bar. (**B**) Bar charts (mean ± SEM with individual data-points, each representing mean of at least 60 cells) showing the expression at the cell surface (HA signal) of WT α_2_δ-1-HA (blue bar) and HA-α_2_δ-1^G209D^ (green bar). Data obtained from six coverslips in two independent experiments, ****P < 0.0001, Student’s *t*-test. (**C**) Western blot experiments for HA-α_2_δ-1 (anti-HA Ab, *top*; molecular weight ∼140–170 kDa for the glycosylated uncleaved and proteolytically cleaved α_2_δ-1 proteins) and GAPDH (used as a control, *bottom*). *Left* panels show whole-cell lysate (WCL) input and *right* panels show cell surface biotinylated samples from tsA-201 cells transfected with HA-α_2_δ-1 WT (lane 1) or HA-α_2_δ-1^G209D^ (lane 2) or empty vector (EV, lane 3). Western blots were performed under reducing conditions, such that the disulphide bonds between α2 and δ were broken. Uncropped blots are in Supplementary Fig. 10A. (**D**) Mean ± SEM and individual data-points of cell surface HA-α_2_δ-1 WT (blue bar) or HA-α_2_δ-1^G209D^ (green bar) measured as a proportion of biotinylated over total protein normalized to HA-α_2_δ-1 WT. **P = 0.0012, Student’s *t*-test. (**E**) Example of whole-cell patch-clamp recordings for Ca_V_2.2-HA co-expressed with β1b and either α_2_δ-1 WT (blue, *left*), empty vector (no α_2_δ, red, centre) or α_2_δ-1^G209D^ (green, *right*). Holding potential −80 mV, steps between −50 and +60 mV for 50 ms (applies to all traces). (**F**) Mean (± SEM) *IV* relationships for the conditions shown in **E**. Ca_V_2.2-HA co-expressed with α_2_δ-1 WT (*n* = 14, blue fileld circles), empty vector (no α_2_δ, *n* = 10, red filled circles) or α_2_δ-1^G209D^ (*n* = 10, green filled circles). The individual and mean data were fit with a modified Boltzmann equation (see ‘Materials and methods’ section). (**G**) G_max_ [nanosiemens (nS)/picofarad (pF)] from the *IV* relationships shown in **F**. Individual data (same symbols as in **F**) and mean ± SEM are plotted. ****P < 0.0001 versus wild-type (one-way ANOVA and Sidak’s *post hoc* test correcting for multiple comparisons).

We then investigated the *in vitro* effect of the p.(Gly209Asp) variant on α_2_δ-1 as a calcium channel subunit. First, we compared cell surface expression of wild-type HA-α_2_δ-1 (HA-α2δ-1 WT) and HA-α_2_δ-1^G20n9D^ in non-permeabilized cells.^5^ As shown by the haemagglutinin (HA) signal, the expression of HA-α_2_δ-1^G209D^ at the cell surface was reduced by ∼80% compared to HA-α_2_δ-1 WT (Fig. 2A and B). In agreement, cell surface biotinylated HA-α_2_δ-1^G209D^ was decreased, by 86.2%, compared to HA-α_2_δ-1 WT (Fig. 2C and D).

### The p.(Gly209Asp) variant abolishes ability of α_2_δ-1 to promote Ca_V_2.2 currents

Ca_V_2.2 currents were then measured in tsA-201 cells transfected with HA-tagged Ca_V_2.2 with β1b and α_2_δ-1 wild-type, α_2_δ-1^G209D^ or no α_2_δ. While α_2_δ-1 wild-type increased Ca_V_2.2 currents by ∼13-fold, α_2_δ-1^G209D^ produced no increase compared to without α_2_δ (Fig. 2E–G). Expression of all subunits was confirmed by western blotting and immunocytochemistry (Supplementary Fig. 6). This effect was not specific to Ca_V_2.2, as α_2_δ-1^G209D^ also did not increase Ca_V_2.1 currents (Supplementary Fig. 7A–C).

**Figure 3:**
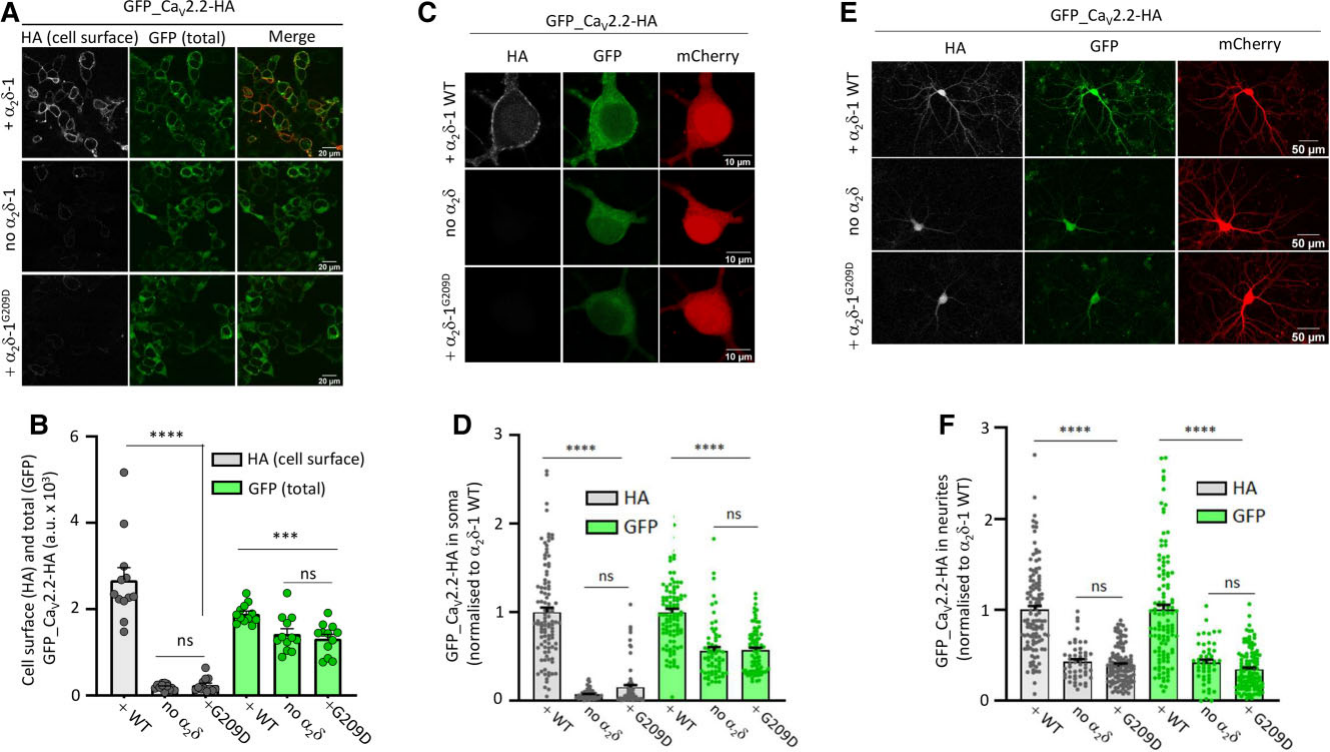
**α_2_δ-1^G209D^ does not enhance Ca_V_2.2 expression at the cell surface in tsA-201 cells and hippocampal neurons**. (**A**) Representative confocal images of tsA-201 cells transfected with GFP_Ca_V_2.2-HA with either α_2_δ-1 wild-type (WT) (without tag; *top row*), empty vector (no α_2_δ, *middle row*) or α_2_δ-1^G209D^ (without tag; *bottom row*). HA staining was performed in non-permeabilized conditions (white, *left*), GFP signal shows total Ca_V_2.2 (*middle*). The right panels represent the merged images (HA is shown in red). Scale bar = 20 μm. (**B**) Bar charts (mean ± SEM with individual data-points representing the mean of at least 50 cells from 12 coverslips) show Ca_V_2.2 expressed at the cell surface (HA signal, grey bars) or total Ca_V_2.2 (GFP, green bars) in the presence of α_2_δ-1 WT, empty vector (no α_2_δ-1) or α_2_δ-1^G209D^. Data obtained from four independent experiments; ns, *P* > 0.05; ***P = 0.0007, ****P < 0.0001, one-way ANOVA and Bonferroni *post hoc* test. (**C**) Representative confocal images of hippocampal somata imaged at ×63 objective and transfected with GFP_Ca_V_2.2-HA and either α_2_δ-1 WT (*top row*), empty vector (no α_2_δ, *middle row*) or α_2_δ-1^G209D^ (*bottom row*) together with β1b and mCherry. HA staining was performed in non-permeabilized conditions (grey, *left*), GFP signal (*middle*) and mCherry (transfection marker, *right*). Scale bar = 10 μm. (**D**) Bar charts (mean ± SEM with individual data-points) of HA (grey bars) and GFP (green bars) for α_2_δ-1 WT (*n* = 102), no α_2_δ (*n* = 63) and α_2_δ-1^G209D^ (*n* = 82). Data obtained from three independent experiments; the values are normalized to α_2_δ-1 WT condition in each experiment. ns, *P* > 0.05; ****P < 0.0001, one-way ANOVA and Bonferroni *post hoc* test. (**E**) Representative confocal images of hippocampal neurons imaged at ×20 objective and transfected with GFP_Ca_V_2.2-HA and either α_2_δ-1 WT (*top row*), empty vector (no α_2_δ, *middle row*) or α_2_δ-1^G209D^ (*bottom row*) together with β1b and mCherry. HA staining was performed in non-permeabilized conditions (grey, *left* panels), GFP signal (*middle*) and mCherry (transfection marker, *right*). Scale bar = 50 μm. (**F**) Bar charts (mean ± SEM with individual data-points) of HA (grey bars) and GFP (green bars) for α_2_δ-1 WT (*n* = 114), no α_2_δ (*n* = 50) and α_2_δ-1^G209D^ (*n* = 117). Data obtained from three independent experiments; the values are normalized to α_2_δ-1 WT condition in each experiment. ns, *P* > 0.05; ****P < 0.0001, one-way ANOVA and Bonferroni *post hoc* test. Note that for **B**, **D** and **F**, total Ca_V_2.2 (GFP) was reduced in both the α_2_δ-1^G209D^ and no α_2_δ conditions compared to α_2_δ-1 WT condition probably because of its increased degradation when Ca_V_2.2 is poorly trafficked out of the endoplasmic reticulum.

We next investigated expression of the calcium channel complex at the plasma membrane, using double-tagged GFP_Ca_V_2.2-HA. When GFP_Ca_V_2.2-HA was co-expressed with α_2_δ-1 wild-type, this resulted in an increase in its cell surface expression compared with no α_2_δ control (Fig. 3A and B). However, this effect was completely absent when Ca_V_2.2 was co-expressed with α_2_δ-1^G209D^ (Fig. 3A and B).

### α_2_δ-1^G209D^ does not promote Ca_V_2.2 cell surface expression or trafficking in hippocampal neurons

Ca_V_2.2 is a neuronal calcium channel, and we therefore investigated the effect of α_2_δ-1^G209D^ on Ca_V_2.2 trafficking in neurons, as previously described.^13^ We first analysed cell surface expression of GFP_Ca_V_2.2-HA in cultured hippocampal cell bodies. As expected, in the presence of α_2_δ-1 WT, GFP_Ca_V_2.2-HA was strongly expressed at the cell surface (HA signal, Fig. 3C and D). In contrast, in the presence of α_2_δ-1^G209D^, GFP_Ca_V_2.2-HA could not be detected at the cell surface, similar to no α_2_δ (Fig. 3C and D).

The neurites of these cells were then imaged. As expected, GFP_Ca_V_2.2-HA showed strong expression when co-expressed with α_2_δ-1 WT; this was observed for both HA (cell surface) and green fluorescent protein (GFP) (total Ca_V_2.2) (Fig. 3E and F). In contrast, α_2_δ-1^G209D^ did not promote trafficking of Ca_V_2.2 into hippocampal neurites (Fig. 3E and F). This is indicated by the finding that both HA (cell surface Ca_V_2.2) and GFP (total Ca_V_2.2) signals were reduced in parallel.

### α_2_δ-1^G209D^ shows reduced complex formation with Ca_V_2.2 and limited proteolytic cleavage

**Figure 4:**
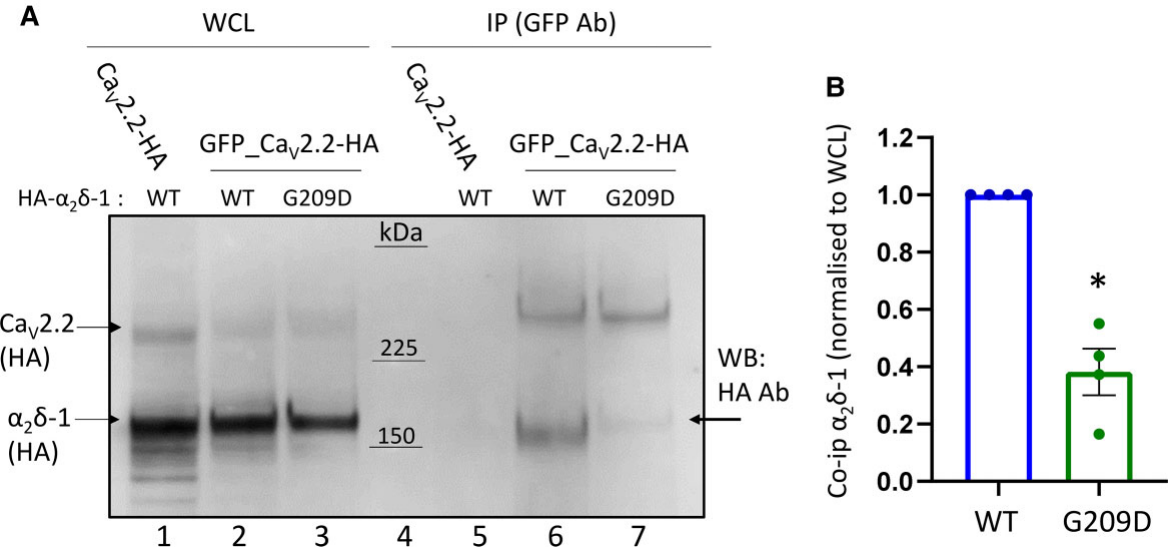
**α_2_δ-1^G209D^ shows reduced interaction with Ca_V_2.2**. (**A**) GFP_Ca_V_2.2-HA was co-expressed with β1b and either HA-α_2_δ-1 WT or HA-α_2_δ-1^G209D^. Both Ca_V_2.2 and α_2_δ-1 proteins were detected in the same western blot using anti-HA antibodies. Immunoblots of whole-cell lysates (WCL) input (*left*) and immunoprecipitated (IP using GFP antibodies) samples (*right*) from tsA-201 cells transfected with Ca_V_2.2-HA, β1b and HA-α_2_δ-1 WT (lanes 1 and 5) or GFP-Ca_V_2.2-HA with either HA-α_2_δ-1 WT (lanes 2 and 6) or HA-α_2_δ-1^G209D^ (lanes 3 and 7). Immunoblots with anti-HA antibody reveal Ca_V_2.2-HA and GFP_Ca_V_2.2-HA (*top* bands) and HA-α_2_δ-1 WT and HA-α_2_δ-1^G209D^ (*lower* bands). Immunoprecipitation was performed with anti-GFP antibody to pull down GFP-Ca_V_2.2-HA and the co-immunoprecipitated α_2_δ-1 is shown below this on the same blot. Ca_V_2.2-HA lacking GFP tag, used as a negative control, shows a lack of co-immunoprecipitation (co-IP) with HA-α_2_δ-1 WT (lane 5). Arrow (lane 7) indicates increased molecular weight of α_2_δ-1^G209D^ relative to α_2_δ-1 WT. Western blots were performed under reducing conditions, such that the disulphide bonds between α_2_ and δ were broken. Uncropped blots are in Supplementary Fig. 10B. (**B**) Mean ± SEM and individual data-points for co-immunoprecipitation of HA α_2_δ-1 WT (blue bar) or α_2_δ-1^G209D^ (green bar) measured as a proportion of total protein (whole-cell lysates) and normalized to HA-α_2_δ-1 WT. *P = 0.0281 (ratio paired *t*-test on non-normalized data).

To examine whether the lack of ability of α_2_δ-1^G209D^ to promote calcium channel function was due to reduced interaction with Ca_V_2.2, we performed co-immunoprecipitation experiments, using GFP_Ca_V_2.2-HA (Fig. 4A). For α_2_δ-1 WT, robust interaction was shown by the presence of HA-α_2_δ-1 WT, co-immunoprecipitated by GFP_Ca_V_2.2-HA, using anti-GFP antibody (Fig. 4A, lane 6). In contrast, very weak co-immunoprecipitation was observed for HA-α2δ-1^G209D^ (Fig. 4A, lane 7), quantified in Fig. 4B. As a control, there was no co-immunoprecipitation of HA-α_2_δ-1 WT using Ca_V_2.2-HA without a GFP tag (Fig. 4A, lane 5).

Interestingly, co-immunoprecipitated HA-α_2_δ-1^G209D^ (Fig. 4A, lane 7, arrow) had a noticeably higher apparent molecular weight compared to HA-α_2_δ-1 WT (lane 6), and we found this was due to almost complete lack of proteolytic cleavage of HA-α_2_δ-1^G209D^ into α_2_ and δ (Supplementary material and Supplementary Fig. 8).

In summary, these results show that α_2_δ-1^G209D^ remains largely as the uncleaved immature form, indicating that it probably remains in the endoplasmic reticulum. In agreement with our previous results for uncleaved α_2_δ-1,^17^ it shows much less complex formation with Ca_V_2.2. This result suggested that α_2_δ-1^G209D^ would be unlikely to interfere with other α_2_δ proteins interacting with Ca_V_2.2. In agreement with this, we found that α_2_δ-1^G209D^ did not affect the ability of α_2_δ-3 to enhance Ca_V_2.2 currents (Supplementary Fig. 9A–C). This result underscores that the p.(Gly209Asp) variant has a loss-of-function effect.

## Discussion

In the current study, we show that biallelic loss-of-function variants in *CACNA2D1* underlie DEE. In Patient 1 the homozygous frameshift variant p.(Ser275Asnfs*13) causes nonsense-mediated mRNA decay of mutated *CACNA2D1* transcripts and absence of α_2_δ-1 in patient-derived fibroblasts. The variants p.(Leu9Alafs*5) and p.(Gly209Asp) in Patient 2 are a combination of a very early frameshift and a missense variant *in trans*, with the latter severely affecting Ca_V_2 calcium channel function.

Our electrophysiological, biochemical and immunocytochemistry data show that α_2_δ-1^G209D^ is completely non-functional, in that, unlike wild-type α_2_δ-1,^5^ it traffics extremely poorly to the cell surface, and does not enhance the function or trafficking of Ca_V_2 channels in both non-neuronal cells and hippocampal neurons. Furthermore, α_2_δ-1^G209D^ shows markedly reduced cleavage into α_2_ and δ, an enzymatic process that normally begins in the Golgi apparatus.^13,17^ This suggests that α_2_δ-1^G209D^ does not traffic beyond the endoplasmic reticulum. Our previous finding that an uncleavable mutant α_2_δ-1 shows lower association with the Ca_V_2.2 α_1_ subunit than the mature cleaved α_2_δ-1,^17^ indicates that the lack of proteolytic cleavage of α_2_δ-1^G209D^ will probably contribute to the observed reduction in interaction of α_2_δ-1^G209D^ with the Ca_V_2.2 α_1_ subunit. This demonstrates the importance of a detailed understanding of α_2_δ-1 processing and function, in order to identify the basis for such deleterious variants.

Variants in *CACNA2D1* have previously been associated with cardiac phenotypes in both humans and mice. Homozygous knockout of *Cacna2d1* in mice resulted in a mild cardiac phenotype and reduced ventricular myocyte calcium current density.^18^ These mice also showed peripheral sensory deficits and delayed development of neuropathic pain-related responses.^19^ Relevant to this, both patients showed insensibility to pain (Table 2). Transgenic mice constitutively over-expressing α_2_δ-1 have no gross nervous system defects.^20^ However, they show spontaneous epileptiform EEG abnormalities and behavioural arrest,^21^ suggesting that not only the spatial and temporal expression but also the expression strength of α_2_δ-1 is critical for proper functioning of the mouse brain. Indeed, α_2_δ-1 is the major α_2_δ isoform in rodent cerebral cortex.^22^ Furthermore, an auto-antibody recognizing α_2_δ-1 is found in cases of autoimmune encephalitis^23^ and amyotrophic lateral sclerosis associated with type 2 diabetes.^24^

In humans, heterozygous variants in *CACNA2D1* have previously been associated with inherited arrhythmogenic disease, including Brugada^25^ and short QT^26^ syndromes, as well as infantile spasms^27^ and intellectual disability and epilepsy.^28^ Re-evaluation of these monoallelic variants, together with genetic data presented here give rise to reasonable doubt about an association of these *CACNA2D1* variants with disease (Supplementary material).

Pathogenic variants in genes encoding several Ca_V_ channels have been associated with neurological diseases in humans, ranging from early-onset severe spinocerebellar ataxia with neurodevelopmental deficits to DEE (see Supplementary material). In *CACNA2D2*, rare biallelic loss-of-function variation has been reported in individuals with DEE, corpus callosum hypoplasia, cerebellar atrophy and ataxia.^9,10^ The two unrelated patients reported here show considerable clinical overlap with individuals carrying homozygous *CACNA2D2* loss-of-function variants, such as global developmental delay and/or intellectual disability, epilepsy and hypoplasia of the corpus callosum. However, atrophy of the brain affects the cerebrum in the two affected individuals with *CACNA2D1* variants, whereas cerebellar atrophy was consistently reported in subjects with *CACNA2D2* variants.^29^ These data indicate that loss of α_2_δ-1 or α_2_δ-2 cannot be compensated by any of the other α_2_δ subunits during development. In agreement with this, important, non-overlapping roles for specific α_2_δ proteins in synapse formation *in vitro* have been identified recently, some of which may be calcium channel-independent.^30^

In conclusion, our data demonstrate that biallelic loss-of-function variants in *CACNA2D1* underlie early-onset DEE characterized by microcephaly, profound developmental delay, seizures, visual impairment, truncal hypotonia, limb spasticity and movement disorder. These clinical features are similar to those in previously reported individuals with homozygous *CACNA2D2* null alleles. Individuals with biallelic *CACNA2D1* or *CACNA2D2* variants all have corpus callosum hypoplasia, while patients with *CACNA2D1* variants show progressive cerebral atrophy whereas subjects harbouring *CACNA2D2* variants have cerebellar atrophy. The loss-of-function frameshift nature of two of the three identified *CACNA2D1* variants, together with our functional studies demonstrating a loss-of-function effect for the amino-acid substitution p.(Gly209Asp), confirm disease causation of homozygous and compound heterozygous *CACNA2D1* variants, while also calling into question a causative role of monoallelic *CACNA2D1* variants in intellectual disability, epilepsy and/or inherited arrhythmogenic diseases.

## Supplementary Material

awac081_Supplementary_Data

## Abbreviations

DEE: developmental and epileptic encephalopathy
GFP: green fluorescent protein
