# Supplementary material for: Biallelic CACNA2D1 loss-of-function variants cause early-onset developmental epileptic encephalopathy
Source: Brain. 2022 Mar 16;145(8):2721–9. doi: 10.1093/brain/awac081 (PMC9420018; doi:10.1093/brain/awac081)
Supplement: awac081_Supplementary_Data [file awac081_supplementary_data.pdf]

Biallelic *CACNA2D1* loss-of-function variants cause early-onset developmental epileptic encephalopathy

Shehrazade Dahimene, Leonie von Elsner, Tess Holling, Lauren S Mattas, Jess Pickard, Davor Lessel, Kjara S Pilch, Ivan Kadurin, Wendy S Pratt, Igor B Zhulin, Hongzheng Dai, Maja Hempel, Maura R Z Ruzhnikov, Kerstin Kutsche and Annette C Dolphin

Supplementary Information

Contents

	Title	Page
Supplementary Methods	Methods	2
Case Reports	Case Reports	9
Discussion	Literature review and discussion of *CACNA2D1* variants	11
Supplementary Table 1	Sequence of oligonucleotides	13
Supplementary Figure 1	*CACNA2D1* variant validation in leukocyte-derived DNA of family 1 and in cDNA obtained from patient 1-derived fibroblasts	14
Supplementary Figure 2	Data set of three independent RT-qPCR experiments	15
Supplementary Figure 3	Full-length immunoblots shown in Fig. 1D	16
Supplementary Figure 4	Determination of *CACNA2D3* mRNA levels in patient-derived fibroblasts	17
Supplementary Figure 5	G209 is invariant in CACNA2D1 and its predecessors	18
Supplementary Figure 6	Immunoblot and immunocytochemistry showing expression of all Ca_V subunits	19
Supplementary Figure 7	$\alpha_2\delta$-1^{G209D} does not promote $Ca_V2.1$ calcium currents	20
Supplementary Figure 8 and associated Results	$\alpha_2\delta$-1^{G209D} shows reduced interaction with $Ca_V2.2$ and limited proteolytic cleavage	21
Supplementary Figure 9 and associated Results	$\alpha_2\delta$-1^{G209D} does not interfere with $\alpha_2\delta$-3 function	23
Supplementary Figure 10	Full-length immunoblots shown in Fig. 2C and 4A and Supplementary Figs. 6 and 8	24
Supplementary References		25

Supplementary Information

Methods

Exome sequencing, sequence data analysis, and variant validation

Genomic DNA was extracted from peripheral blood samples using standard procedures. We performed trio exome sequencing (trio ES) with DNA samples of patient 1 and both healthy parents as described previously [1]. Briefly, coding DNA fragments were enriched with a SureSelect Human All Exon 50Mb V5 Kit (Agilent), and captured libraries were then loaded on a HiSeq2500 platform (Illumina). Reads were aligned to the human reference genome (UCSC GRCh37/hg19) using the Burrows-Wheeler Aligner (BWA, v.0.5.87.5), and detection of genetic variation was performed with SAMtools (v.0.1.18), PINDEL (v. 0.2.4t), and ExomeDepth (v.1.0.0). Sanger sequencing of leukocyte-derived DNA obtained from patient 1 and his parents permitted *CACNA2D1* variant validation (NM_000722.3) and segregation analysis. Primer sequences are described in **Supplementary Table 1**.

For Patient 2, trio ES was undertaken with DNA samples of the proband and both healthy parents at Baylor Genetics, as described previously [2]. Briefly, coding DNA fragments were enriched with VCRome 2.1 in-solution exome probes (Roche NimbleGen), and captured libraries were then loaded on a HiSeq2000 platform (Illumina). Data analysis and interpretation were performed by the Baylor Genetics analytics pipeline.

The Patient 2 missense variant c.626G>A/p.(Gly209Asp) was predicted to be damaging by the following *in silico* tools CADD (29.7)[3], M-CAP (0.026)[4], REVEL (0.685)[5], and Meta-SNP (0.814)[6]. *CACNA2D1* is highly intolerant of functional genetic variation, as only 59% of the expected missense (observed/expected score: 0.59) and 15% of the expected loss-of-function variants (observed/expected score: 0.15) are found in gnomAD (v.2.1.1)[7]. Sanger sequencing of fibroblast-derived DNA obtained from patient 2 permitted *CACNA2D1* variant validation (NM_000722.3). Primer sequences are in **Supplementary Table 1**.

Bioinformatic analysis of CACNA2D1 homologs

BLAST searches [8] against the National Center for Biotechnology Information (NCBI) RefSeq database were carried out, with default parameters, using the human CACNA2D1 sequence (NP_000713.2) as a query. In this analysis, the $\alpha_2\delta$ proteins are denoted CACNA2D1-4. The resulting set contained 5000 sequences (including isoforms) that were aligned using the

LINS-I method in MAFFT program [9] and edited in Jalview [10] to discard incomplete sequences, trim the alignment, and reduce redundancy (100% identical sequences). A neighbour-joining tree was constructed from this dataset using the MEGA X package [11]. The tree topology was then compared to a previously published trees of CACNA2D1, CACNA2D2 and their homologs [12], to identify orthologs, paralogs and CACNA2D1/CACNA2D2 ancestors in the retrieved dataset. CACNA2D2-like, CACNA2D3, and CACNA2D4 sequences were then removed from the final alignment.

Cell culture

Primary fibroblasts obtained from a skin biopsy of patient 1 and four controls were cultured in Dulbecco's modified Eagle medium (DMEM; Thermo Fisher Scientific) supplemented with 10% fetal bovine serum (FBS; GE Healthcare) and penicillin-streptomycin (100 U/ml and 100 µg/ml, respectively; Thermo Fisher Scientific) and incubated at 37 °C in a humidified atmosphere with 5% CO_2. Cells were confirmed as mycoplasma-free by PCR testing. Primer sequences are described in **Supplementary Table 1**.

tsA-201 cells (European Collection of Cell Cultures, female sex) were plated onto cell culture flasks or coverslips coated in poly-L-lysine, and cultured in DMEM supplemented with fetal bovine serum (5 %), Penicillin-Streptomycin (1 %) and GlutaMAX (Thermo Fisher Scientific, 1 %), in a 5 % CO_2 incubator at 37 °C.

Hippocampal neurons were obtained from P0/P1 rat pups (Sprague-Dawley, both sexes). All experiments were performed in accordance with the UK Home Office Animals (Scientific procedures) Act 1986, using a Schedule 1 method, with UCL ethical approval. Briefly, hippocampi were dissected, chopped into small pieces and treated for 40 min at 37 °C with a papain solution containing: 70 units /ml of papain, 0.2 mg/ml L-cysteine, 0.2 mg/ml bovine serum albumin (BSA), 1000 units/ml DNase-1 and 5 mg/ml glucose (all from Sigma Aldrich) in Hank's basal salt solution medium (Thermo Fisher Scientific). Hippocampi were then washed with inactivation medium (Minimum Essential Medium (MEM); Thermo Fisher Scientific, 5% FBS, 0.38 % glucose, 0.25% BSA) and mechanically dissociated with a P1000 micropipette with a polypropylene plastic tip in serum medium (MEM, 5 % FBS, 1.38 % glucose). After centrifugation at 1000 rpm for 10 min, cells were resuspended in serum medium and seeded at a concentration of approximately 4000 cells/mm^2 onto coverslips precoated with poly-D-lysine hydrobromide (Sigma; 50 µg/ml). 1 h later, cells were covered with serum-free neuronal plating medium comprising Neurobasal Medium, supplemented with B27 (2 %), GlutaMAX (1 %) and 1 unit/ml penicillin, 1 µg/ml streptomycin (all from

Thermo Fisher Scientific). Neurons were kept in a 5 % CO_2 incubator at 37 °C and half the medium was replaced every 3-4 days. At 7 days *in vitro* and 2 h before transfection, half of the medium was removed, and fresh medium was added.

Antibodies

The following antibodies and dilutions were used: anti-hemagglutinin (HA, rat, Sigma-Aldrich), anti- Glyceraldehyde 3-phosphate dehydrogenase (GAPDH, mouse, clone 6C5), anti-green fluorescent protein (GFP, 1:200, rabbit polyclonal, Living Colors), mouse monoclonal anti-$\alpha_2\delta$-1 (1:500, Sigma Aldrich D219, clone 20A), rabbit polyclonal anti-$\alpha_2\delta$-1 (1:1,000, Proteintech, #27453-1-AP), mouse monoclonal anti-GAPDH (1:5,000; Abcam; ab8245; clone 6C5), rabbit anti Cav2.2 II-III loop [13], rabbit anti-β antibody (2491) [14] and mouse monoclonal anti β1b (1:250) [15]. For immunoblotting, secondary goat anti-rat Horseradish Peroxidase (HRP; Biorad), donkey anti-rabbit HRP (1:7,500, Amersham, #NA934V), goat anti-mouse HRP (Biorad and 1:5,000, NA931V, GE Healthcare) were used. For immunocytochemistry, secondary anti-rat-Alexa Fluor 594, anti-rat-Alexa Fluor 488, anti-rat-Alexa Fluor 647 and anti-Rabbit Alexa Fluor 674 antibodies (1:500, Life Technologies) were used as appropriate.

Immunoblot analysis

Whole-cell lysates from patient and control fibroblasts were prepared, and immunoblotting was performed as previously described [16]. Briefly, cells were harvested in ice-cold RIPA buffer (50 mM Tris-HCl pH 8.0, 150 mM NaCl, 1% NP-40, 0.5% DOC, and 0.1% SDS) supplemented with Mini Protease Inhibitor (Roche), protein extracts were separated on sodium dodecyl sulfate-polyacrylamide gel electrophoresis (SDS-PAGE) and transferred to polyvinylidene fluoride membranes. Membranes were blocked followed by incubation with the indicated primary antibody overnight at 4 °C and by HRP-linked secondary antibody at room temperature for 1 h or with fluorescent dye-linked anti-tubulin antibody at room temperature for 1h. Chemiluminescent and fluorescent signals were digitally imaged using a ChemiDoc MP (Bio-Rad). Band intensities were determined with the Image Lab v6.0 software (Bio-Rad).

Expression constructs and mutagenesis

The following expression constructs were used: Cav2.2 (rabbit, D14157.1), from which were derived Cav2.2 HA [17], GFP_Cav2.2 [18] and GFP_Cav2.2-HA [19], Cav2.1 (rat), β1b (rat) [20], $\alpha_2\delta$-1 (rat, M86621.1, termed WT in this study), from which was derived HA-$\alpha_2\delta$-1 WT [21]. The

G209D variant was introduced into rat $\alpha_2\delta$-1 by standard PCR-based techniques, changing the specific nucleotide codon GGC to GAC. The HA-tagged $\alpha_2\delta$-1^{G209D} construct was created by subcloning from HA-$\alpha_2\delta$-1 WT. All the constructs were verified by Sanger sequencing (Source Bioscience). All cDNAs were used in either pcDNA3 vector for expression in tsA-201 cells or pCAGGS for hippocampal neurons. Empty pcDNA3 or pCAGGS vectors were used as controls where stated; mCherry [22] and CD8 [23] were transfection markers where stated.

Cell transfection

The tsA-201 cells were transfected with PolyJet (unless otherwise stated) in a 3:1 ratio with DNA mix, according to the manufacturer's protocol. Following incubation overnight at 37 °C, culture medium was changed. When observing cell surface expression of $\alpha_2\delta$-1 alone, the transfection mix consisted of plasmids containing cDNAs encoding HA-$\alpha_2\delta$-1 WT, HA-$\alpha_2\delta$-1^{G209D} or empty vector, with mCherry in a 6:1 ratio. For experiments including $Ca_V2.2$, the transfection mix consisted of plasmids containing cDNAs encoding GFP_$Ca_V2.2$-HA or GFP_$Ca_V2.2$, β1b and either $\alpha_2\delta$-1 WT, α2δ-1^{G209D} or empty vector in a ratio of 3:2:2. For co-IP experiments, transfection mixes of plasmids encoding the following cDNAs, GFP_$Ca_V2.2$-HA, β1b and either HA-$\alpha_2\delta$-1 WT, HA-$\alpha_2\delta$-1^{G209D} or empty vector. For electrophysiological experiments, $Ca_V2.2$-HA or $Ca_V2.1$ was co-expressed with β1b and either $\alpha_2\delta$-1 WT, $\alpha_2\delta$-1^{G209D} or empty vector in a ratio of 3:2:2. To test if $\alpha_2\delta$-1^{G209D} affects $\alpha_2\delta$-3 function, $Ca_V2.2$-HA was co transfected with β1b and $\alpha_2\delta$-3, together with either empty vector or $\alpha_2\delta$-1^{G209D} in a ratio 3:2:1:1. CD8 was used as a transfection marker. For cell surface biotinylation, expression constructs used were either HA-$\alpha_2\delta$-1 WT, HA-$\alpha_2\delta$-1^{G209D} or empty vector. tsA-201 cells were transfected with Fugene6 in a 3:1 ratio with plasmids, according to the manufacturer's protocol and incubated at 37 °C for 48 h.

Hippocampal neurons were transfected with Lipofectamine 2000 (Life Technology), according to the manufacturer's protocol. The transfection mix consisted of plasmids containing cDNAs encoding GFP_$Ca_V2.2$-HA, β1b and either $\alpha_2\delta$-1 WT, $\alpha_2\delta$-1^{G209D} or empty vector and mCherry in a ratio of 3:2:2:1.

Electrophysiology

$Ca_V2.1$ and $Ca_V2.2$ currents in transfected tsA-201 cells were investigated by whole cell patch clamp recording[24]. The patch pipette solution contained in mM: Cs-aspartate, 140; EGTA, 5; $MgCl_2$, 2; $CaCl_2$, 0.1; K_2ATP, 2; HEPES, 10; pH 7.2, 310 mOsm with sucrose. The external solution for recording Ba^{2+} currents contained in mM: tetraethylammonium (TEA) Br, 160; KCl, 3; $NaHCO_3$, 1.0; $MgCl_2$, 1.0; HEPES, 10; glucose, 4; $BaCl_2$, 1, pH 7.4, 320 mosM with

sucrose. 1 mM extracellular Ba^{2+} was the charge carrier. Pipettes of resistance 2–4 MΩ were used. An Axopatch 1D or Axon 200B amplifier was used, and whole cell voltage-clamp recordings were sampled at 10 kHz frequency, filtered at 2 kHz and digitized at 1 kHz. 70–80% series resistance compensation was applied and all recorded currents were leak subtracted using P/8 protocol. Analysis was performed using Pclamp 9 (Molecular Devices) and Origin 7 (Microcal Origin, Northampton, MA). To obtain the maximum conductance (G_{max}), Current-voltage (*IV)* relationships were fitted by a modified Boltzmann equation as follows: $I=G_{max}*(V-V_{rev})/(1+exp(-(V-V_{50,\,act})/k))$ where I is the current density (in pA/pF), G_{max} is in nS/pF, V_{rev} is the apparent reversal potential, $V_{50,\,act}$ is the midpoint voltage for current activation, and k is the slope factor.

Cell surface biotinylation and immunoblotting

Cell surface biotinylation was performed as described previously [25]. Briefly, 48 h after transfection, tsA-201 cells were incubated for 30 min at room temperature with 0.5 mg/ml Premium Grade EZ-link Sulfo-NHS-LC-Biotin (Thermo Scientific) in PBS and the reaction was quenched with 200 mM glycine. The cells were resuspended in PBS, pH 7.4 at 4 °C containing 1 % Igepal; 0.1 % SDS and protease inhibitors (PI, cOmplete, Roche), to allow cell lysis, cleared by centrifugation at 18,000 × g and assayed for total protein (Bradford assay, Biorad). Biotinylated lysates (equalized to between 0.5 and 1 mg/ml total protein concentration) were applied to 40 µl prewashed streptavidin-agarose beads (Pierce High Capacity, Thermo Scientific) and rotated overnight at 4 °C. The beads were then washed 3 times with PBS containing 0.1 % Igepal. The samples containing precipitated cell surface protein fractions and whole cell lysate (WCL) corresponding to 20 – 40 µg total protein were mixed with Laemmli sample buffer supplemented with 100 mM dithiothreitol (reducing agent), resolved by SDS-PAGE on 3 – 8 % Tris-Acetate (Invitrogen) and transferred to polyvinylidene fluoride (PVDF) membrane (Biorad). The signal was obtained by HRP reaction with fluorescent product (ECL 2; Thermo Scientific) and membranes were scanned on a Typhoon 9410 phosphorimager (GE Healthcare). Quantification was performed using ImageJ.

Co-immunoprecipitation

Co-immunoprecipitation (co-IP) was performed as described previously [25]. A tsA-201 cell pellet derived from one confluent 75 cm^2 flask was resuspended in co-IP buffer (20 mM HEPES (pH 7.4), 300 mM NaCl, 1 % Digitonin and PI), sonicated for 8 s at 20 kHz and rotated for 1 h at 4 °C. The samples were then diluted with an equal volume of 20 mM

HEPES (pH 7.4), 300 mM NaCl, 1 mM $CaCl_2$, 1 mM $MgCl_2$, with PI (to 0.5 % final concentration of Digitonin), mixed by pipetting and centrifuged at 18,000 x g for 20 min. The supernatants were collected and assayed for total protein (Bradford assay; Biorad). 2 mg of total protein was adjusted to 3 mg/ml with co-IP buffer and incubated overnight at 4 °C with anti-GFP polyclonal antibody (1:200; BD Biosciences). 30 μl A/G PLUS Agarose slurry prewashed in co-IP buffer (Santa Cruz) was added to each tube and further rotated for 2 h at 4 °C. The beads were then washed three times with co-IP buffer containing 0.2% Digitonin. When required, the beads were resuspended in an equal volume of PBS, pH 7.4 containing 1 % Triton X-100; 0.1 % SDS and protease inhibitors (PI, cOmplete, Roche) and proteins were deglycosylated with PNGase-F for 3 h at 37 °C. Aliquots of the initial WCL prior to co-immunoprecipitation were also deglycosylated in parallel; 2 × Laemmli buffer with 100 mM dithiothreitol was added and samples were analysed by SDS-PAGE and western blotting as described above. Where indicated, the membrane was re-blotted, by washing with Tris-buffered saline 0.5% Igepal and it was then re-probed with primary antibodies followed by the appropriate secondary antibodies coupled with HRP.

Immunocytochemistry

Cells were fixed with 4 % paraformaldehyde in phosphate-buffered saline (PBS, pH 7.4) for 5 min, when indicated, the cells were permeabilised with Triton X100 0.2% for 5 min. The cells were incubated with blocking buffer (20 % goat serum, 4 % BSA in PBS) for 1 h at room temperature before being incubated with primary antibodies diluted in 0.5 x blocking buffer for 1 h at room temperature. For hippocampal neurons, rat anti-HA antibody incubation was carried out at 4 °C overnight. After washing, samples were incubated with secondary antibodies, anti-rat Alexa Fluor 488, anti-rat Alexa Fluor 594, anti-rat Alexa Fluor 647 or anti-Rabbit Alexa Fluor 674 at a dilution of 1:500 for 1 h at room temperature. Coverslips were washed and mounted in VectaShield (Vector Laboratories). Imaging was performed on Zeiss LSM 780 confocal microscope.

Image analysis

The tsA-201 cell images were obtained using a 63 × objective at a resolution of 1024 × 1024 pixels and an optical section of 0.8–1 μm. After choosing a region of interest containing transfected cells (mCherry- or GFP-positive cells), the 3 × 3 tile function of the microscope allowed imaging of a larger area selected without bias. Images were then analyzed using Fiji. Surface labelling (HA staining) was measured using a freehand line tool of 5 μm width and manually tracing the surface of the cells. Total protein staining (GFP signal) was measured by

drawing around the cell (omitting the nucleus). The value of the mean pixel intensity in different channels was measured separately and background was subtracted by measuring the intensity of an imaged area without transfected cells.

In order to measure the percentage of cells that express all the three subunits, tsA-201 cells were transfected with GFP_Ca_V2.2 together with β1b and HA $\alpha_2\delta$-1 WT or HA $\alpha_2\delta$-1^{G209D}. Intracellular region of interests were placed in the merged image and the mean pixel intensity in the three channels was measured separately and background (signal from un-transfected cells) was subtracted. This procedure was repeated in at least 7 images and total number of analysed cells was approximately 160 cells.

Hippocampal neurons were imaged using a 20 × objective with a 5 µm optical section. The fluorescence intensity along neuronal projections was assessed as follows: two concentric circles of 100 µm and 150 µm diameter were drawn around each neuronal cell body. A freehand line tool of 5 µm width tracing the neuronal processes (3 to 5 per neuron) between the circles was drawn in the mCherry images and used as template for GFP and HA images. Hippocampal somata were imaged at 63 x objective with a 1 µm optical section, surface labelling (HA staining) was measured using a freehand line tool (5 µm width) and manually tracing the surface of the cell body and total protein staining (GFP signal) was measured by drawing around the cell (omitting the nucleus). The mean pixel intensity in the different channels were measured, the background was subtracted and the data were normalised to the control for each experiment.

Data analysis, statistics and availability

Quantitative data are presented by GraphPad Prism 8 software (v8.0.0; GraphPad Software, Inc.) or Origin-Pro 2021 as the mean ± standard error of the mean (SEM) with individual data points. Student's t-test and one-way analysis of variance (ANOVA) followed by a Bonferroni or other stated *post hoc* test for multiple comparisons were used. A p value < 0.05 was considered statistically significant (* $p \leq 0.05$; ** $p \leq 0.01$; *** $p \leq 0.001$; **** $p \leq 0.0001$). Data supporting the findings of this study are available from the corresponding authors, upon reasonable request

Case Reports

Patient 1 is the only child of his healthy parents who are first-degree cousins (**Tables 1 and 2**). A paternal uncle had a developmental and epileptic encephalopathy with onset in infancy. There is no additional significant family history. He was born at 37 weeks gestation via normal spontaneous vaginal delivery after an uncomplicated pregnancy. APGAR scores were 10, 10 and birth measurements were in the normal range, with weight of 2960 g (-0.5 z), length of 50 cm (-0.2 z), and occipitofrontal head circumference (OFC) of 33 cm (-1.0 z). He came to our attention at 3 months of age due to severe hypotonia and poor eye contact. By 6 months of age, he was noted to be microcephalic (41 cm, -2.1 z), with height and weight within the normal range (7.2 kg, -0.6 z; 70 cm, 0.7 z). He was noted to have severe global hypotonia and delayed gross motor development with poor head control. He did not fix or follow objects, and did not grasp or transfer objects from hand to hand. There was minimal reaction to visual stimuli, but he did respond to sound, such as his mother's voice. Extended metabolic work up revealed normal results. Brain MRI at 7 months of age showed increased ventricular size and extra axial spaces as well as hypoplasia of the corpus callosum (**Fig. 1A**). EEG was normal. Absence seizures were first suspected clinically at 9 months of age. At 13 months he developed feeding difficulties with failure to thrive, severe sleep disturbance, and notable nystagmus bilaterally. He developed generalized seizures at 19 months of age in additional to absence seizures. A gastrostomy tube was placed at 4 years of age. At his last examination at 4 years 11 months, he was microcephalic with an OFC of 48.9 cm (-2.1 z), and weight and length were within normal range. He was dysmorphic with bitemporal narrowing, medial flaring of eyebrows, and an open mouth with tented upper lip. Neurological exam was notable for severe truncal muscular hypotonia with poor head control, spasticity in all extremities, and dystonic and choreiform movements. He had made little neurodevelopmental progress without any major milestones having been achieved. He was not able to grasp, sit, or roll over. Speech development was severely impaired: he vocalized but did not speak. He did not respond to visual stimuli, but response to sound was apparently normal. MRI of the brain at 4 years and 10 months showed a hypoplastic corpus callosum and progressive frontotemporal and mesial temporal atrophy (**Fig. 1A**).

Patient 2 is the youngest child of healthy, nonconsanguineous parents (**Tables 1 and 2**). He had two older brothers, one of whom carries the diagnoses of an attention deficit hyperactivity disorder and autistic features requiring behavioral therapy. There is no additional significant family history. He was born at term (40 weeks) via spontaneous vaginal delivery after an unremarkable pregnancy. His birth weight was 3345 g (-0.73z). Additional birth

measurements and APGAR scores are not known. He was noted to be "floppy" since birth, however when hospitalized with a respiratory syncytial virus infection at 2.5 months, his neurological examination was recorded as normal. He was first evaluated by neurology at 7 months of age, at which time he was noted to have global hypotonia, and was not yet able to roll or sit. Also noted were poor eye contact and minimal interaction with his environment as well as brisk reflexes. He had feeding difficulties with frequent choking prompting referral for consideration of a gastrostomy tube, which was ultimately placed at 13 months of age. He was evaluated by the neurogenetics clinic initially at 11 months of age, where he continued to have severe global hypotonia, global developmental delays and also noted were near-constant distal hyperkinetic movements and orofacial dyskinesias. His examination also demonstrated evolving spasticity in his limbs with back arching episodes (opisthotonic posturing) when upset. Shortly afterwards, at 11.5 months of age he had the onset of epilepsy, with his initial EEG suggestive of a developmental and epileptic encephalopathy with diffuse encephalopathy and multifocal epileptiform discharges. Since that time, his epilepsy has evolved to include focal (hemi-clonic) seizures with impaired awareness, atypical absences, and more recently his EEG during sleep has met criteria for electrical status epilepticus in sleep. Antiseizure medications trialed include levetiracetam, topiramate, and valproic acid. He has had the best response from valproic acid, and is currently on monotherapy for his epilepsy and clonazepam for his movement disorder. MRI of the brain at 11 months and then 26 months showed generalized volume loss that progressed and borderline thinning of the corpus callosum, no additional structural abnormalities, besides an incidental pars intermedia cyst (**Fig. 1B**). At last follow-up at 4 years of age, general examination was notable for microcephaly (OFC: 47.5 cm/-2.02z) with posterior plagiocephaly, mild bitemporal narrowing, low-set and larger appearing ears, mild bilateral ptosis, facial hypotonia with his mouth held open, a high-arched palate, widely spaced and blunted teeth due to frequent bruxism, and small hands and feet (toddler 7 US shoe, EU 23). Neurological examination was notable for encephalopathy with absent verbal speech (reportedly he can say 5-10 words) and inability to follow simple commands, intermittent eye contact with disconjugate gaze, severe global hypotonia with spasticity at the knees and elbows. He was unable to sit independently and could not bear weight when held in a standing position. There were frequent small amplitude complex hyperkinetic movements of his distal extremities, predominantly of his hands. In terms of his genetic work-up, karyotype, chromosomal microarray, methylation analysis for Prader-Willi and Angelman syndrome and both epilepsy and neurometabolic multi-gene panels were non-diagnostic.

Literature review and discussion of *CACNA2D1* variants

As stated in the Discussion, pathogenic variants in genes encoding several voltage-gated calcium channels have been associated with neurological diseases in humans. These disease genes comprise *CACNA1A, CACNA1B, CACNA1E, CACNA1G* and *CACNA1I,* all coding for Ca_V2 and Ca_V3 α1 subunits[26-30]. Pathogenic *CACNA1S* variants are associated with diseases of skeletal muscle function[31].

CACNA2D1 is one of several genes included in 7q21.11 microdeletions reported in individuals with intellectual disability with or without epilepsy[32-35]. The smallest region of overlap of the deletions contains the three genes *HGF*, *CACNA2D1*, and *PCLO* suggesting that haploinsufficiency of one of these genes is primarily underlying the phenotype[35]. However, this study did not detect possible pathogenic single nucleotide variants in any of the three genes individually, in 4,293 patients with undiagnosed developmental disorders[35]. Recently, two *de novo*, possibly truncating *CACNA2D1* variants have been reported as disease-causing alterations in the literature[36,37]. The c.2625delT / p.(Asn876Ilefs*37) (originally reported as p.V875fs, but corrected herein) variant was reported in a 13-year-old girl with normal intelligence. At the age of 6 months, she developed infantile spasms and had hypsarrhythmia on electroencephalogram (EEG). Spasms responded to adrenocorticotropic hormone, and she was seizure-free without antiepileptic treatment at age 13 years[37]. The second patient, a 14-year-old female with intellectual disability, epilepsy, and craniofacial dysmorphism carried an intronic 1-bp insertion c.659-2_659-1insT that has not been further investigated at the mRNA level[36]. Phenotypic variability in the two reported patients, especially the presence or absence of intellectual disability, and the presence of a several heterozygous *CACNA2D1* likely loss-of-function variants in the gnomAD and LOVD databases (https://databases.lovd.nl/shared/genes/CACNA2D1) bring into question the pathogenicity of the described monoallelic *CACNA2D1* variants.

In humans, heterozygous missense variants in *CACNA2D1* have previously been associated with inherited arrhythmogenic disease, including Brugada[38] and short QT[39] syndromes. These include the variants p.(Asp550Tyr), p.(Ser709Asn), p.(Ser755Thr), p.(Gln917His), and p.(Ser956Tyr). Genetic and clinical re-evaluation of four of the five variants indicated p.(Ser709Asn) and p.(Gln917His) to be likely benign and of uncertain clinical significance, respectively[40,41]. The functional consequences of the variants p.(Asp550Tyr) and p.(Ser709Asn) have been investigated, showing largely non-significant reduction in function

with respect to $Ca_V1.2$ current enhancement[42]. We re-evaluated the p.(Ser755Thr), p.(Gln917His) and p.(Ser956Tyr) variants. p.(Ser755Thr) has a worldwide MAF of 0.08% and was found in the homozygous state in two individuals in the gnomAD database, a finding that seriously questions an association of the heterozygous variant with short QT syndrome. p.(Gln917His) and p.(Ser956Tyr) are rare variants as they have a worldwide MAF of 0.01% and 0.001%, respectively. However, three *in silico* programs we are routinely using to predict pathogenicity of missense variants predict p.(Gln917His) (CADD: 14; REVEL: 0.125; Meta-SNP: 0.314) and p.(Ser956Tyr) (CADD: 23; REVEL: 0.163; Meta-SNP: 0.120) not to have a severe impact on $\alpha_2\delta$-1 function. These data together with the lack of replication studies reporting additional possible pathogenic *CACNA2D1* variants in individuals with arrhythmia syndromes give rise to reasonable doubt about an association of the aforementioned heterozygous *CACNA2D1* missense variants with Brugada and short QT syndrome. In addition, the healthy parents of both patients reported here are heterozygous carriers of a *CACNA2D1* null allele demonstrating that monoallelic loss-of-function variants in this gene are neither highly penetrant variants for intellectual disability and/or epilepsy nor cardiac arrhythmia.

Supplementary Table 1: Sequence of oligonucleotides

CACNA2D1 primers for variant validation in family 1			
**Template**	**Intron**	**Direction**	**Sequence (5' → 3')**
genomic DNA (leukocytes)	9	forward	GGT CAG TGC ATA CAT TAT CTT TCC
	10	reverse	GCA AAT TTA ATT CAG GAA AAT GC
**Template**	**Exon**	**Direction**	**Sequence (5' → 3')**
cDNA (fibroblasts)	9	forward	GAC ATG CTT ATT CTG GTG GAT G
	12	reverse	CCT CCA TCC GTG AAT AGC AT
***CACNA2D1* primers for variant validation in patient 2**			
**Template**	**Intron**	**Direction**	**Sequence (5' → 3')**
genomic DNA (leukocytes)	5'UTR	forward	GCG TGT GCT GCT CTT CCT
	1	reverse	GGC CGA TGA GCA AAG ATT G
	6	forward	CCA GTC AAA CTG TCC TCT AAA GTG
	7	reverse	CAG AAA CAT TGA GGC TGG AAC
**Template**	**Exon**	**Direction**	**Sequence (5' → 3')**
cDNA (fibroblasts)	5'UTR	forward	GCG TGT GCT GCT CTT CCT
	3	reverse	TCC CTG GCT GCA ATT TCT AC
	6	forward	AGG CAG CCA GAG GAT AAA AC
	8	reverse	TTG GAG TTC TAC TAT TAT CAA CC
**Primer sequences for RT-qPCR on cDNA derived from fibroblasts**			
**Gene**	**Exon**	**Direction**	**Sequence (5' → 3')**
CACNA2D1	6	forward	CTA CTG ACA TCT ATG AGG GCT CAA C
	7	reverse	CAC TGC CAA AAA CCT GCC AC
	10	forward	ACC CTC TCA GAT GAT GAT TTC GTG
	11	reverse	GCT GTT CAA AAG CAA AAC TAA AGC C
CACNA2D3	2	forward	TTC CCA GCT TCT GCA AAA GAA ATA C
	3	reverse	GCC TCA GAC TTC TTG TGA AAC ATC
	9	forward	GCA AGC CGA CAG GAC AAA CAA AG
	11	reverse	TCC ACC GCC CCA TCA GTT ATG AG
GAPDH	3	forward	TGA CCC CTT CAT TGA CCT CAA C
	5	reverse	GCA TCG CCC CAC TTG ATT TTG
**Primer sequences for mycoplasma detection by PCR**			
**Template**	**Direction**		**Sequence (5' → 3')**
Cell culture supernatant	forward		GGG AGC AAA CAG GAT TAG ATA CCC T
	reverse		TGC ACC ATC CTG TCA CTC TGT TAC CCT C

Supplementary Results and Figures

Supplementary Fig. 1: *CACNA2D1* variant validation in DNA of family 1 and patient 2 and in fibroblast-derived cDNA of patients 1 and 2

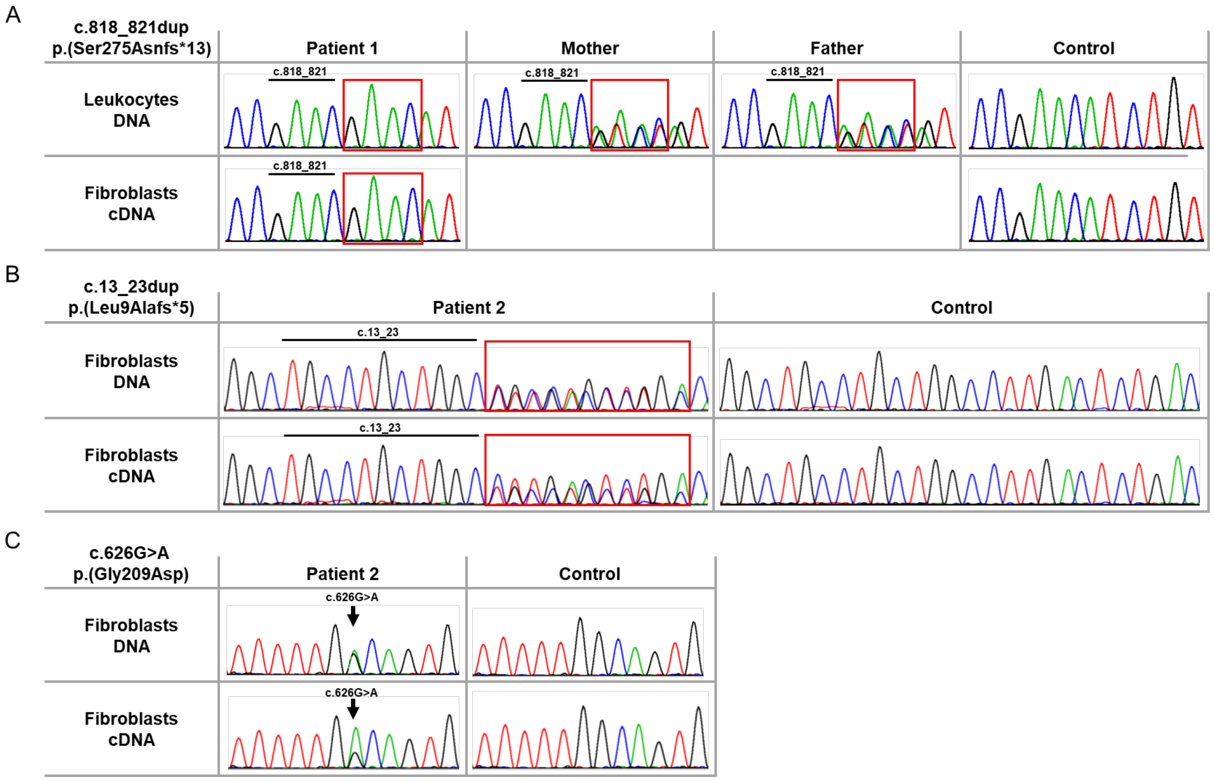

(A) Partial sequence electropherograms showing the *CACNA2D1* c.818_821dup variant in leukocyte-derived DNA and fibroblast-derived cDNA of patient 1 and leukocyte-derived DNA of his mother and father. Patient 1 is homozygous for the 4-bp duplication (red box) and his healthy parents are heterozygous carriers. For control purposes, leukocyte-derived DNA and fibroblast-derived cDNA of a healthy individual (control) were used and respective sequence traces are shown on the right. (B and C) Partial sequence electropherograms showing the *CACNA2D1* variants c.13_23dup / p.(Leu9Alafs*5) (red box) (B) and c.626G>A / p.(Gly209Asp) (arrow) (C) in fibroblast-derived DNA in the heterozygous state and in fibroblast-derived cDNA of patient 2. Respective *CACNA2D1* wild-type sequences of fibroblast-derived DNA and cDNA (control) are shown on the right in B and C.

Supplementary Fig. 2: Dataset of three independent RT-qPCR experiments (for Fig. 1C)

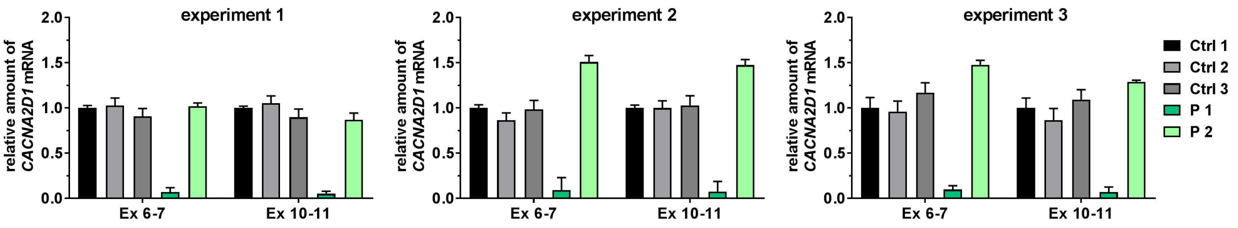

Quantification of relative *CACNA2D1* transcript levels in fibroblasts of patient 1 and 2 and three healthy individuals (controls 1-3) by RT-qPCR using two *CACNA2D1*-specific primer pairs generating amplicons for exons 6-7 and 10-11. *GAPDH* mRNA was used as an internal control; the amount of *CACNA2D1* mRNA relative to *GAPDH* mRNA is presented. Mean ± SEM of technical triplicates from three independent experiments are shown. Ctrl: control; P: patient.

Supplementary Fig. 3: Full-length immunoblots shown in Fig. 1D

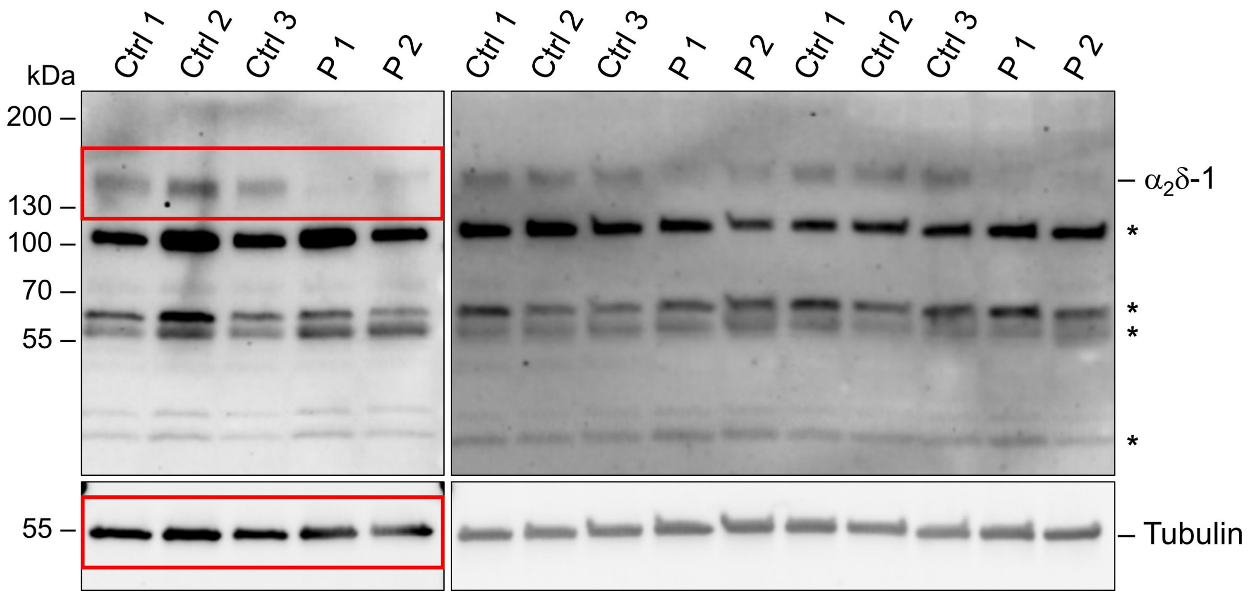

Full-length immunoblots of whole-cell lysates obtained from patient- and control-derived fibroblasts of three different passages are shown. $\alpha_2\delta$-1 was detected by immunoblotting using an anti-$\alpha_2\delta$-1 antibody. Anti-tubulin antibody was used to demonstrate equal loading. Non-specific bands are marked by asterisks. Red boxes indicate the cropped area.

Supplementary Fig. 4: Determination of *CACNA2D3* mRNA levels in patient and control-derived fibroblasts

A

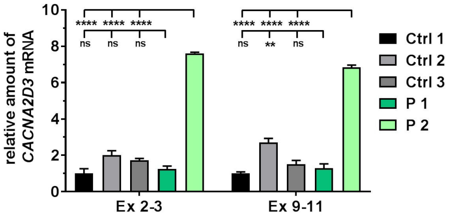

B

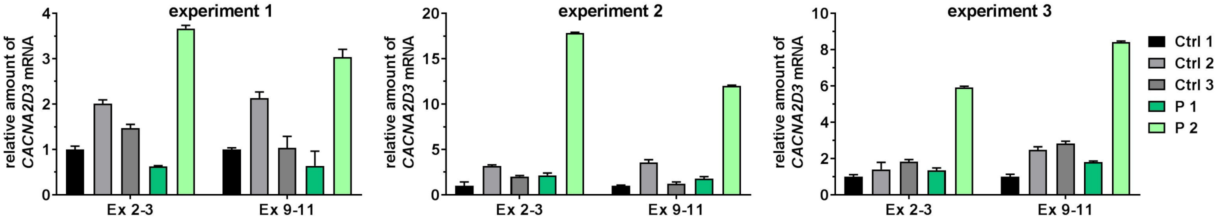

(A and B) Relative quantification of *CACNA2D3* transcripts by RT-qPCR using two *CACNA2D3*-specific primer pairs generating amplicons for exons 2-3 and 9-11. RNA was obtained from fibroblasts of patient 1 and 2 and three healthy individuals (controls 1 to 3). *GAPDH* mRNA was used as an internal control; and the amount of target mRNA relative to *GAPDH* mRNA is presented. (A) Mean ± SEM of three independent experiments, each performed in technical triplicate, is shown. One-way ANOVA with Bonferroni post hoc test for multiple comparisons was used for statistical analysis: ns, $P > 0.05$; ** $P = 0.0011$; **** $P < 0.0001$. (B) Mean ± SEM of technical triplicates from three independent experiments are shown. Ctrl: control; ns: not significant; P: patient.

Supplementary Fig. 5: G209 is invariant in CACNA2D1 and its predecessors.

		209	
Human	NP_000713.2	SLLWQVFGSATGLAR	(202-216)
Chicken	NP_001170793.2	TLLWQVFGSATGLAR	(203-217)
Frog	NP_001090738.1	TLLWQVFGSATGLAR	(203-217)
Shark	XP_038667399.1	TLMWQVFGSATGLAR	(213-227)
Zebrafish 1	NP_001038425.2	SLHWQVFGSATGLAR	(203-217)
Zebrafish 2	XP_017207543.2	TLLWQVFGSATGLAR	(210-224)
Lancelet	XP_019633684.1	GLLWQYFGSSKGPFR	(208-222)
Acorn worm	XP_006820803.1	SLIWQYFGSAEGYMR	(183-197)
Sea urchin	XP_030828832.1	QLLWQYFGSADGFYR	(194-208)
Brachiopod	XP_023931087.1	NLLWQYFGGQKGFLR	(74-88)
Tunicate	XP_002126163.2	GTRWMFVGTNTGVFR	(219-233)
Nematode	NP_001022364.1	EMGHQYIGTYSGLTR	(184-198)
Sponge	XP_019850671.1	RVTSQYLGSVTGVYR	(202-216)

In this analysis, the gene products are denoted with non-italicised gene names. A segment of multiple sequence alignment of representative CACNA2D1 homologs is shown. All sequences from vertebrates are CACNA2D1 orthologs, except for two CACNA2D1 paralogs from Zebrafish, and all sequences from invertebrates are CACNA2D1/CACNA2D2 ancestors [12]. The position corresponding to G209 in a human CACNA2D1 gene product is highlighted in yellow. NCBI accession numbers are provided for each sequence.

These data indicate that all changes in this position were selected against, during approximately 600 million years of evolution. Consequently, we conclude that any change in the G209 position is likely to be deleterious to CACNA2D1 function. For comparison, some of the nearby positions in the protein are tolerant to various substitutions, e.g. position A211 tolerates changes to small, large, polar, aliphatic, and aromatic residues.

Supplementary Fig. 6: Immunoblot and immunocytochemistry showing expression of all Ca_V subunits

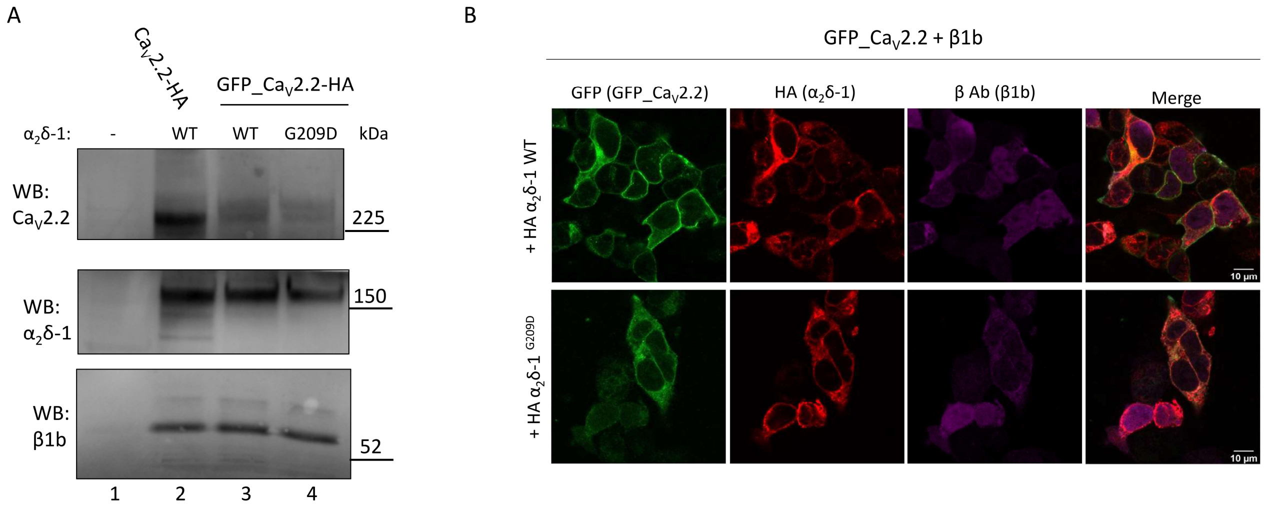

(

A) Example immunoblot analysis showing the expression of $Ca_V2.2$ and the auxiliary subunits β1b and $\alpha_2\delta$-1. tsA-201 cells were transfected with $Ca_V2.2$-HA (lane 2) or GFP_$Ca_V2.2$-HA (lanes 3 and 4), plus β1b, and either HA-$\alpha_2\delta$-1 WT (lanes 2 and 3) or HA-$\alpha_2\delta$-1^{G209D} (lane 4). Lane 1 is a negative control condition (-: untransfected cells). The WCL membrane from Fig. 4 was reblotted with the following antibodies: anti-$Ca_V2.2$ II III loop (top panel), anti-$\alpha_2\delta$-1 (middle panel) and anti-β1b (bottom panel). Full length blots are in Supplementary Fig. 10C.

(B) Confocal images of tsA-201 cells transfected with GFP_$Ca_V2.2$, β1b and either HA $\alpha_2\delta$-1 WT (top row) or HA $\alpha_2\delta$-1^{G209D} (bottom row). HA (HA Ab) and β (β2491 Ab) staining was performed in permeabilised conditions, GFP signal shows total $Ca_V2.2$. The right panels represent the merged images with the 10 µm scale bar. Quantification showed that 98.7 % and 93.2 % of transfected cells expressed all subunits $Ca_V2.2$, β1b and either HA-α2δ-1 WT or HA-α2δ-1^{G209D}, respectively.

Supplementary Fig. 7: α2δ-1^{G209D} does not promote Cav2.1 calcium currents.

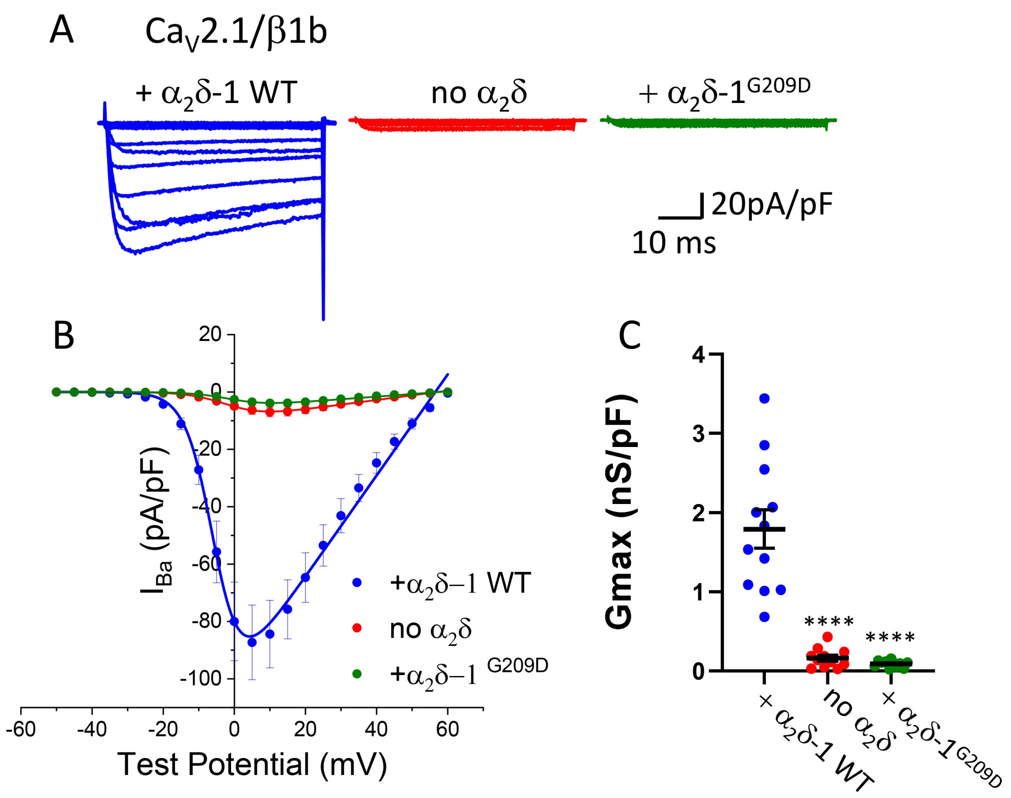

(A) Example of whole-cell patch-clamp recordings for Cav2.1 co-expressed with β1b and either α2δ-1 WT (left), empty vector (no α2δ, centre) or α2δ-1^{G209D} (right). Holding potential −80 mV, steps between −50 and +60 mV for 50 ms (applies to all traces).

(B) Mean (± SEM) *IV* relationships for the conditions shown in (A). Cav2.1 co-expressed with α2δ-1 WT (*n* = 12, blue solid circles), empty vector (no α2δ, *n* = 11, red solid circles) or α2δ-1^{G209D} (*n* = 11, green solid circles). The individual and mean data were fit with a modified Boltzmann equation (see Methods).

(C) G_{max} (*nS/pF*) from the *IV* relationships shown in (E). Individual data (same symbols as in B) and mean ± SEM are plotted. **** $P < 0.0001$ (one-way ANOVA and Sidak's *post hoc* test correcting for multiple comparisons).

Supplementary Fig. 8: $\alpha_2\delta$-1^{G209D} shows reduced interaction with Cav2.2 and limited proteolytic cleavage

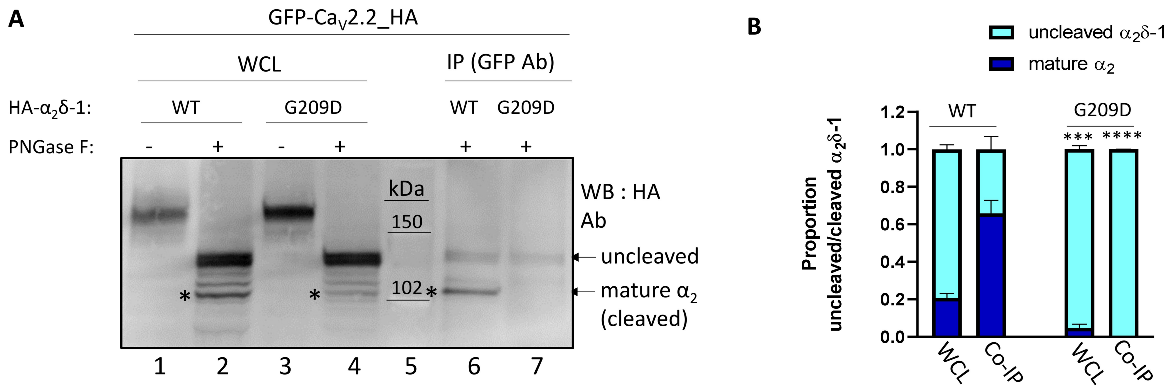

Supplementary Results

We hypothesized that the observed increase in MW of $\alpha_2\delta$-1^{G209D} compared to $\alpha_2\delta$-1 WT (**Fig. 4**), could be due to reduced cleavage of $\alpha_2\delta$-1^{G209D} relative to $\alpha_2\delta$-1 WT. The $\alpha_2\delta$-1 protein is encoded by a single gene that is post-translationally cleaved into α_2 and δ moieties[43], and we have identified previously that this cleavage occurs during $\alpha_2\delta$-1 trafficking, probably commencing in the Golgi complex[25,44]. Because of extensive glycosylation of $\alpha_2\delta$-1, it was necessary to deglycosylate the samples with PNGase F, in order to accurately determine their molecular weights (Supplementary **Fig. 8A**). In the WCLs, deglycosylated samples clearly showed partial cleavage of $\alpha_2\delta$-1 (Supplementary **Fig. 8A**, lanes 2 and 4 compared to lanes 1 and 3, respectively). Furthermore, the amount of mature fully-cleaved α_2 band (indicated by the star) was reduced for $\alpha_2\delta$-1^{G209D} compared to $\alpha_2\delta$-1 WT (Supplementary **Fig. 8A,** lane 4 compared to lane 2), and this was quantified in Supplementary **Fig. 8B**. In the WCL, the percentage of cleaved $\alpha_2\delta$-1 was 21.0 ± 0.11 % and 5.0 ± 0.09 % for $\alpha_2\delta$-1 WT and $\alpha_2\delta$-1^{G209D}, respectively.

We have previously shown that the mature cleaved form of $\alpha_2\delta$-1 (α_2-1) co-immunoprecipitates with Ca$_V$2.2 to a greater extent than uncleaved $\alpha_2\delta$-1[45]. In agreement with this, in the present study we also found that there was more mature cleaved $\alpha_2\delta$-1 WT co-immunoprecipitated with Ca$_V$2.2 than the proportion of $\alpha_2\delta$-1 WT in the WCL (Supplementary **Fig. 8A,** lane 6 and Supplementary **Fig. 8B**). In contrast, $\alpha_2\delta$-1^{G209D} showed very little proteolytic cleavage (Supplementary **Fig. 8A,**

lane 4), and that which was co-immunoprecipitated with $Ca_V2.2$ was exclusively the uncleaved form (Supplementary **Fig. 8A,** lane 7 and Supplementary **Fig. 8B**).

It should be noted that the lack of proteolytic cleavage of $\alpha_2\delta$-1^{G209D} is not responsible per se for its reduced trafficking, as $\alpha_2\delta$-1 with a mutation in the proteolytic cleavage site is still able to reach the plasma membrane[25]. Rather $\alpha_2\delta$-1^{G209D} is likely misfolded and retained in the endoplasmic reticulum (ER).

The reduction of $\alpha_2\delta$-1 in Patient 2 fibroblasts to somewhat below the expected 50% level (Fig. 1), is possibly due to ER-associated protein degradation (ERAD) as the experiments shown here provide evidence that $\alpha_2\delta$-1^{G209D} protein is retained in the ER, and thus shows reduced proteolytic cleavage.

Legend to Supplementary Fig. 8

(A) Co-immunoprecipitation of cleaved (HA-α_2-1) and uncleaved (HA-$\alpha_2\delta$-1) moieties of HA-$\alpha_2\delta$-1 WT and HA-$\alpha_2\delta$-1^{G209D} with $Ca_V2.2$**.** Immunoblots of WCL input (lanes 1 - 4) and co-IP (lanes 6 and 7) from tsA-201 cells transfected with GFP_$Ca_V2.2$, together with HA-$\alpha_2\delta$-1 WT (lanes 1, 2 and 6) or HA-$\alpha_2\delta$-1^{G209D} (lanes 3, 4 and 7). Immunoblots of WCL with anti-HA antibody show glycosylated (lanes 1 and 3) and deglycosylated (treated with PNGase-F, lanes 2 and 4) HA-$\alpha_2\delta$-1 (MW ~140 kDa for uncleaved $\alpha_2\delta$-1); the star (*) indicates mature proteolytically cleaved α_2-1 (~105 kDa). Immunoprecipitation of PNGase-F-treated, deglycosylated HA-$\alpha_2\delta$-1 WT (lane 6) or HA-$\alpha_2\delta$-1^{G209D} (lane 7). The arrows indicate uncleaved $\alpha_2\delta$-1 and mature cleaved α_2. Full length blots are in Supplementary Fig. 10D

(B) Proteolytic cleavage of $\alpha_2\delta$-1 expressed as ratio of cleaved (mature α_2, dark blue) to uncleaved $\alpha_2\delta$-1 (light blue), calculated for input WCL and for co-IP fraction. Mean ± SEM of four independent experiments. Comparing cleaved $\alpha_2\delta$-1 WT and $\alpha_2\delta$-1^{G209D}, *** $P = 0.0010$ for WCL and **** $P < 0.0001$ for co-IP (Student's t-test).

Supplementary Fig. 9: $\alpha_2\delta$-1^{G209D} does not interfere with $\alpha_2\delta$-3 function.

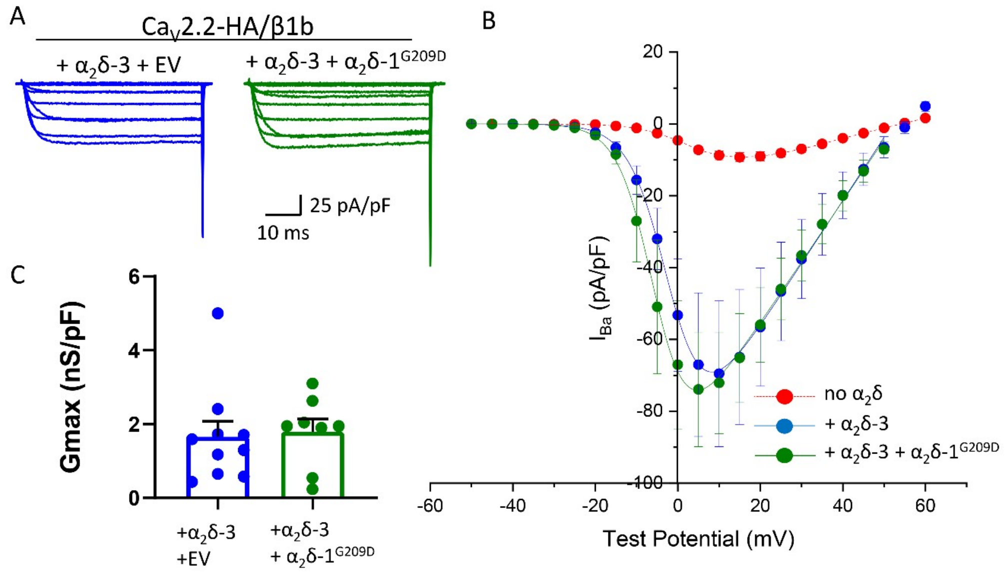

Supplementary Results

We found that in patient 2, but not patient 1, the fibroblast *CACNA2D3* mRNA level was increased several-fold (Supplementary Fig. 4). Because non-overlapping roles for specific $\alpha_2\delta$ proteins in synapse formation have been described [46-48], we wished to examine whether, if this were replicated at the protein level in brain tissue, $\alpha_2\delta$-3 function would be affected by the presence of the $\alpha_2\delta$-1^{G209D} protein, and found no interference with $\alpha_2\delta$-3 function (Supplementary Fig. 9A-C).

Legend to Supplementary Fig. 9

(A) Example of whole-cell patch-clamp recordings for Ca_V2.2-HA co-expressed with β1b and either $\alpha_2\delta$-3 and empty vector (EV, left) or $\alpha_2\delta$-3 and $\alpha_2\delta$-1^{G209D} (right). Holding potential −80 mV, steps between −50 and +60 mV for 50 ms (applies to all traces).

(B) Mean (± SEM) current-voltage relationships for the conditions shown in (A). Ca_V2.2-HA co-expressed with $\alpha_2\delta$-3 and empty vector (n = 10, blue solid circles) or $\alpha_2\delta$-3 and $\alpha_2\delta$-1^{G209D} (n=8, green solid circles). Control condition (no $\alpha_2\delta$, red circles and dashed line) data are replotted from **Fig. 2F** for comparison. Mean data were fit with a modified Boltzmann equation (see Methods).

(C) G_{max} (nS/pF) from the current-voltage relationships shown in (B). Individual data (same symbols as in B) and mean ± SEM are plotted. ns, p=0.81 (Student's t test).

Supplementary Fig. 10: Full-length immunoblots for data shown in Figs. 2C and 4A, and Supplementary Figures 6 and 8.

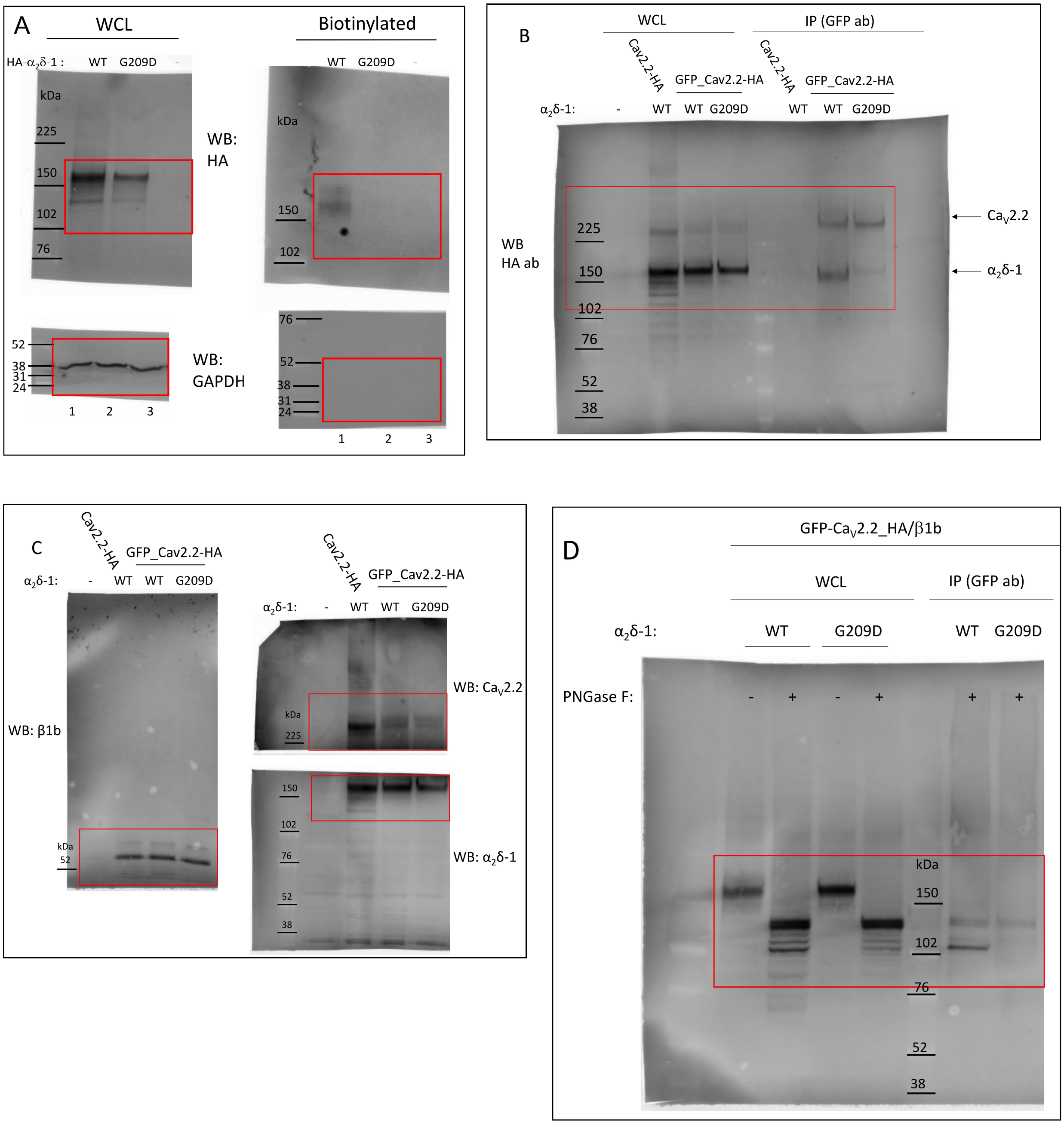

Full length immunoblots for (A) Fig. 2C; (B) Fig. 4A; (C) Supplementary Fig. 6; (D) Supplementary Fig. 8. Note that in some cases, as illustrated, blots were cut prior to immunoblotting for reasons of economy or to reveal multiple proteins on the same gel. Red boxes indicate the approximate cropped area used.

References

1. Coste de Bagneaux P, von Elsner L, Bierhals T, *et al*. A homozygous missense variant in CACNB4 encoding the auxiliary calcium channel beta4 subunit causes a severe neurodevelopmental disorder and impairs channel and non-channel functions. *PLoS Genet*. 2020;16:e1008625.
2. Yang Y, Muzny DM, Reid JG, *et al*. Clinical whole-exome sequencing for the diagnosis of mendelian disorders. *N Engl J Med*. 2013;369:1502-11.
3. Kircher M, Witten DM, Jain P, O'Roak BJ, Cooper GM, Shendure J. A general framework for estimating the relative pathogenicity of human genetic variants. *Nat Genet*. 2014;46:310-5.
4. Jagadeesh KA, Wenger AM, Berger MJ, *et al*. M-CAP eliminates a majority of variants of uncertain significance in clinical exomes at high sensitivity. *Nat Genet*. 2016;48:1581-1586.
5. Ioannidis NM, Rothstein JH, Pejaver V, *et al*. REVEL: An Ensemble Method for Predicting the Pathogenicity of Rare Missense Variants. *Am J Hum Genet*. 2016;99:877-885.
6. Capriotti E, Altman RB, Bromberg Y. Collective judgment predicts disease-associated single nucleotide variants. *BMC Genomics*. 2013;14 Suppl 3:S2.
7. Karczewski KJ, Francioli LC, Tiao G, *et al*. The mutational constraint spectrum quantified from variation in 141,456 humans. *Nature*. 2020;581:434-443.
8. Altschul SF, Madden TL, Schaffer AA, *et al*. Gapped BLAST and PSI-BLAST: a new generation of protein database search programs. *Nucleic Acids Res*. 1997;25:3389-402.
9. Katoh K, Rozewicki J, Yamada KD. MAFFT online service: multiple sequence alignment, interactive sequence choice and visualization. *Brief Bioinform*. 2019;20:1160-1166.
10. Procter JB, Carstairs GM, Soares B, *et al*. Alignment of Biological Sequences with Jalview. *Methods Mol Biol*. 2021;2231:203-224.
11. Kumar S, Stecher G, Li M, Knyaz C, Tamura K. MEGA X: Molecular Evolutionary Genetics Analysis across Computing Platforms. *Mol Biol Evol*. 2018;35:1547-1549.
12. Gumerov VM, Andrianova EP, Matilla MA, *et al*. Amino acid sensor conserved from bacteria to humans. *PNAS (USA)*. 2022;in press:
13. Raghib A, Bertaso F, Davies A, *et al*. Dominant-negative synthesis suppression of voltage-gated calcium channel $Ca_v2.2$ induced by truncated constructs. *Journal of Neuroscience*. 2001;21:8495-8504.
14. Berrow NS, Campbell V, Fitzgerald EG, Brickley K, Dolphin AC. Antisense depletion of β-subunits modulates the biophysical and pharmacological properties of neuronal calcium channels. *Journal of Physiology (Lond)*. 1995;482:481-491.
15. Bogdanov Y, Brice NL, Canti C, *et al*. Acidic motif responsible for plasma membrane association of the voltage-dependent calcium channel β1b subunit. *European Journal of Neuroscience*. 2000;12:894-902.
16. Schneeberger PE, von Elsner L, Barker EL, *et al*. Bi-allelic Pathogenic Variants in HS2ST1 Cause a Syndrome Characterized by Developmental Delay and Corpus Callosum, Skeletal, and Renal Abnormalities. *Am J Hum Genet*. 2020;107:1044-1061.
17. Cassidy JS, Ferron L, Kadurin I, Pratt WS, Dolphin AC. Functional exofacially tagged N-type calcium channels elucidate the interaction with auxiliary alpha2delta-1 subunits. *Proc Natl Acad Sci U S A*. 2014;111:8979-8984.
18. Page KM, Rothwell SW, Dolphin AC. The CaVbeta subunit protects the I-II loop of the voltage-gated calcium channel, CaV2.2, from proteasomal degradation but not oligo-ubiquitination. *J Biol Chem*. 2016;291:20402-20416.
19. Macabuag N, Dolphin AC. Alternative splicing in CaV2.2 regulates neuronal trafficking via adaptor protein complex-1 adaptor protein binding motifs. *Journal of Neuroscience*. 2015;35: 14636–14652.
20. Pragnell M, Sakamoto J, Jay SD, Campbell KP. Cloning and tissue-specific expression of the brain calcium channel β-subunit. *FEBS Letters*. 1991;291:253-258.

21. Kadurin I, Alvarez-Laviada A, Ng SF, *et al*. Calcium currents are enhanced by alpha2delta-1 lacking its membrane anchor. *J Biol Chem*. 2012;287:33554-66.
22. Shaner NC, Campbell RE, Steinbach PA, Giepmans BN, Palmer AE, Tsien RY. Improved monomeric red, orange and yellow fluorescent proteins derived from Discosoma sp. red fluorescent protein. *Nat Biotechnol*. 2004;22:1567-72.
23. Rougier JS, van Bemmelen MX, Bruce MC, *et al*. Molecular determinants of voltage-gated sodium channel regulation by the Nedd4/Nedd4-like proteins. *Am J Physiol Cell Physiol*. 2005;288:C692-701.
24. Dahimene S, Page KM, Kadurin I, *et al*. The $\alpha_2\delta$-like Protein Cachd1 Increases N-type Calcium Currents and Cell Surface Expression and Competes with $\alpha_2\delta$-1. *Cell Reports*. 2018;25:1610–1621.
25. Kadurin I, Ferron L, Rothwell SW, *et al*. Proteolytic maturation of α2δ represents a checkpoint for activation and neuronal trafficking of latent calcium channels *ELife*. 2016;5: e21143.
26. Epi KC. De Novo Mutations in SLC1A2 and CACNA1A Are Important Causes of Epileptic Encephalopathies. *Am J Hum Genet*. 2016;99:287-98.
27. Gorman KM, Meyer E, Grozeva D, *et al*. Bi-allelic Loss-of-Function CACNA1B Mutations in Progressive Epilepsy-Dyskinesia. *Am J Hum Genet*. 2019;104:948-956.
28. Chemin J, Siquier-Pernet K, Nicouleau M, *et al*. De novo mutation screening in childhood-onset cerebellar atrophy identifies gain-of-function mutations in the CACNA1G calcium channel gene. *Brain*. 2018;141:1998-2013.
29. Helbig KL, Lauerer RJ, Bahr JC, *et al*. De Novo Pathogenic Variants in CACNA1E Cause Developmental and Epileptic Encephalopathy with Contractures, Macrocephaly, and Dyskinesias. *Am J Hum Genet*. 2018;103:666-678.
30. El Ghaleb Y, Schneeberger PE, Fernandez-Quintero ML, *et al*. CACNA1I gain-of-function mutations differentially affect channel gating and cause neurodevelopmental disorders. *Brain*. 2021;
31. Flucher BE. Skeletal muscle CaV1.1 channelopathies. *Pflugers Arch*. 2020;472:739-754.
32. Mefford HC, Yendle SC, Hsu C, *et al*. Rare copy number variants are an important cause of epileptic encephalopathies. *Ann Neurol*. 2011;70:974-85.
33. Vergult S, Dheedene A, Meurs A, *et al*. Genomic aberrations of the CACNA2D1 gene in three patients with epilepsy and intellectual disability. *Eur J Hum Genet*. 2015;23:628-632.
34. Mazzaschi RL, Ashton F, Aftimos S, George AM, Love DR. Implications of a Chr7q21.11 Microdeletion and the Role of the PCLO Gene in Developmental Delay. *Sultan Qaboos Univ Med J*. 2013;13:306-10.
35. Siddique A, Willoughby J, Study DDD, McNeill A. A 7q21.11 microdeletion presenting with apparent intellectual disability without epilepsy. *Am J Med Genet A*. 2017;173:1128-1130.
36. Valentino F, Bruno LP, Doddato G, *et al*. Exome Sequencing in 200 Intellectual Disability/Autistic Patients: New Candidates and Atypical Presentations. *Brain Sci*. 2021;11:
37. Hino-Fukuyo N, Kikuchi A, Arai-Ichinoi N, *et al*. Genomic analysis identifies candidate pathogenic variants in 9 of 18 patients with unexplained West syndrome. *Hum Genet*. 2015;134:649-58.
38. Burashnikov E, Pfeiffer R, Barajas-Martinez H, *et al*. Mutations in the cardiac L-type calcium channel associated with inherited J-wave syndromes and sudden cardiac death. *Heart Rhythm*. 2010;7:1872-1882.
39. Templin C, Ghadri JR, Rougier JS, *et al*. Identification of a novel loss-of-function calcium channel gene mutation in short QT syndrome (SQTS6). *Eur Heart J*. 2011;32:1077-1088.
40. Risgaard B, Jabbari R, Refsgaard L, *et al*. High prevalence of genetic variants previously associated with Brugada syndrome in new exome data. *Clin Genet*. 2013;84:489-95.
41. Campuzano O, Sarquella-Brugada G, Fernandez-Falgueras A, *et al*. Genetic interpretation and clinical translation of minor genes related to Brugada syndrome. *Hum Mutat*. 2019;40:749-764.
42. Bourdin B, Shakeri B, Tetreault MP, Sauve R, Lesage S, Parent L. Functional characterization of CaValpha2delta mutations associated with sudden cardiac death. *J Biol Chem*. 2015;290:2854-2869.

43. De Jongh KS, Warner C, Catterall WA. Subunits of purified calcium channels. $\alpha2$ and δ are encoded by the same gene. *Journal of Biological Chemistry*. 1990;265:14738-14741.
44. Kadurin I, Rothwell SW, Lana B, Nieto-Rostro M, Dolphin AC. LRP1 influences trafficking of N-type calcium channels via interaction with the auxiliary alpha2delta-1 subunit. *Sci Rep*. 2017;7:43802.
45. Ferron L, Kadurin I, Dolphin AC. Proteolytic maturation of alpha2delta controls the probability of synaptic vesicular release. *Elife*. 2018;7:e37507.
46. Bikbaev A, Ciuraszkiewicz-Wojciech A, Heck J*, et al*. Auxiliary alpha2delta1 and alpha2delta3 Subunits of Calcium Channels Drive Excitatory and Inhibitory Neuronal Network Development. *J Neurosci*. 2020;40:4824-4841.
47. Geisler S, Schopf CL, Stanika R*, et al*. Presynaptic alpha2delta-2 Calcium Channel Subunits Regulate Postsynaptic GABAA Receptor Abundance and Axonal Wiring. *J Neurosci*. 2019;39:2581-2605.
48. Schöpf CL, Ablinger C, Geisler SM*, et al*. Presynaptic alpha2delta subunits are key organizers of glutamatergic synapses. *Proc Natl Acad Sci U S A*. 2021;118:
